# Ecological resilience in ulcerative colitis: microbial dynamics of donor and resident species in a longitudinal fecal microbiota transplantation study

**DOI:** 10.1093/ismeco/ycaf119

**Published:** 2025-07-16

**Authors:** Susanne Pinto, Elisa Benincà, Sam Nooij, Elisabeth M Terveer, Josbert J Keller, Andrea E van der Meulen – de Jong, Ewout W Steyerberg, Johannes A Bogaards

**Affiliations:** Department of Biomedical Data Sciences, Leiden University Medical Center, Einthovenweg 20, 2333 ZC Leiden, the Netherlands; Centre for Infectious Disease Control, National Institute for Public Health and the Environment (RIVM), Antonie van Leeuwenhoeklaan 9, 3721 MA Bilthoven, the Netherlands; Leiden University Center for Infectious Diseases (LUCID) Research, Leiden University Medical Center, Albinusdreef 2, 2333 ZG Leiden, the Netherlands; Leiden University Center for Infectious Diseases (LUCID) Research, Leiden University Medical Center, Albinusdreef 2, 2333 ZG Leiden, the Netherlands; Netherlands Donor Feces Bank, LUCID Medical Microbiology & Infection Control, Leiden University Medical Center, Albinusdreef 2, 2333 ZG Leiden, the Netherlands; Netherlands Donor Feces Bank, LUCID Medical Microbiology & Infection Control, Leiden University Medical Center, Albinusdreef 2, 2333 ZG Leiden, the Netherlands; Department of Gastroenterology and Hepatology, Leiden University Medical Center, Albinusdreef 2, 2333 ZG Leiden, the Netherlands; Department of Gastroenterology, Haaglanden Medisch Centrum, Bronovolaan 5, 2597 AX The Hague, the Netherlands; Department of Gastroenterology and Hepatology, Leiden University Medical Center, Albinusdreef 2, 2333 ZG Leiden, the Netherlands; Department of Biomedical Data Sciences, Leiden University Medical Center, Einthovenweg 20, 2333 ZC Leiden, the Netherlands; Department of Epidemiology and Data Science, Amsterdam UMC location Vrije Universiteit, Van der Boechorststraat 7, 1081 BT Amsterdam, the Netherlands; Amsterdam Institute for Immunology & Infectious Diseases (AI&I), Amsterdam UMC, De Boelelaan 1089a, 1081 HV Amsterdam, the Netherlands

**Keywords:** gastrointestinal microbiome, ecological succession, species dynamics

## Abstract

Fecal microbiota transplantation (FMT) is a promising treatment for the chronic immune-mediated disease ulcerative colitis (UC). However, the microbial dynamics underlying clinical remission remain poorly understood. To investigate these dynamics, we analysed data from 22 UC patients treated with four rounds of FMT donated by two healthy donors. Microbiota samples from patients were collected at nine timepoints before, during, and after treatment, covering a period of 14 weeks. Additionally, 27 donor samples were analysed. Species in the recipients’ gut microbiota were categorised into ecological categories based on their origin and temporal dynamics: species already present in the recipient pre-FMT, species derived from the donor, or novel species, i.e. absent before FMT in both recipient and donor but detected during or after treatment. Overdispersed Poisson regression models were employed to model the number of species within each category over time. Furthermore, we investigated the change in relative abundance for recipient, colonising, and novel species. The results revealed that recipient species with higher relative abundances prior to FMT were more likely to persist following FMT. Notably, patients who achieved combined clinical and endoscopic remission at week 14 retained a higher number of recipient species compared to non-responders. In contrast, non-responders initially exhibited colonisation of more donor species than responders, but colonisation rate decreased over time in non-responders whereas colonisation rate remained stable in responders. These findings suggest that clinical remission following FMT is associated with controlled incorporation of donor species without replacement of resident species, which may reflect a resilient recipient gut community.

## Introduction

Fecal microbiota transplantation (FMT) is the transfer of fecal matter, including gut microorganisms, from a healthy donor to a diseased recipient to modulate the recipient’s disturbed gut microbiota [1–3]. FMT is effective in recurrent *Clostridioides difficile* infection [2, 3], but success rate is lower for more complex diseases, such as Inflammatory Bowel Disease (IBD) [4, 5]. A possible cause for this reduced efficacy is the tendency of the recipient’s microbiota to revert to its pre-FMT adverse state [6]. Transition to a healthier state is likely helped by colonisation of donor-derived microorganisms. Therefore, it has been suggested that the success of FMT depends on the donor’s gut microbial diversity and composition [7, 8]. The extent to which shifts in the patient’s microbiota towards the donor’s microbiota are beneficial for resolving gut microbiota disturbances remains unclear [6, 9–11]. This donor-centric view has been challenged, and the importance of the recipient and procedural factors to determine FMT outcomes has been highlighted [12–15].

In previous analyses of the FMT trial for ulcerative colitis (UC), involving patients treated with four rounds of FMT donated by two healthy donors, we examined the colonisation of specific microbial species following FMT, and their associations with clinical remission [11, 16]. Longitudinal models and cluster analysis suggested that FMT success in UC patients was associated with Prevotellaceae control, while increased Lachnospiraceae and Ruminococcaceae abundances coincided with clinical remission [11]. The donors harbored high abundances of Clostridiaceae, Bifidobacteriaceae, Ruminococcaceae, and Lachnospiraceae and low abundances of Prevotellaceae [16]. Interestingly, we observed that the rate of microbial engraftment did not correlate with clinical remission [16], and gut microbiota composition did not transition to resemble that of the donors at follow-up [11]. In *β*-diversity analysis using Aitchison distance, samples from the more successful donor clustered closer to responders than to non-responders, but still formed a cluster separate from patient samples [11]. Moreover, of the two healthy donors, the one with the more successful transfers had a lower *α*-diversity [16].

Similarly, a meta-analysis conducted by Schmidt et al. [12] involving 316 FMT procedures across various disease indications, including IBD, highlighted the importance of donor-recipient microbiome complementarity in shaping treatment outcomes [12]. In their study, clinical success was not correlated with donor strain colonisation or replacement of recipient species. Instead, recipient factors seemed to play a more important role in determining FMT outcomes than donor-related factors [12]. The seemingly limited role of donor species colonisation in predicting clinical outcome of FMT defies the “super-donor” hypothesis and necessitates deeper investigation into the ecological changes underlying clinical remission.

In this study, the role of donor and recipient microbial species in determining clinical outcome of FMT is investigated further by applying the conceptual framework introduced by Schmidt et al. [12] to a longitudinal setting. We capitalize on a randomized controlled trial [16] with dense repeated sampling to map the succession dynamics in the recipient’s gut microbiota of UC patients following FMT treatment in relation to clinical remission. Our analysis focuses on ecological dynamics at species level, categorising all taxa based on their origin and temporal presence: already present in the recipient before FMT, derived from the donor, or detected during or after the FMTs while absent in both the pre-FMT recipient and the donor.

## Subjects and methods

### The study population

A total of 24 adult patients experiencing mild to moderate exacerbations of UC were included in a double-blind randomized controlled trial at LUMC [16]. Following pretreatment with either budesonide (n = 12) or placebo (n = 12), patients received four fecal transplants at weekly intervals from one of the two donors (D07 and D08) [17]. Two patients did not complete all four FMTs and were excluded from analysis. Demographic variables and subject characteristics are provided in Supplementary Table S1, with further details provided by van Lingen et al. [16] and in Supplementary Information S1.

Stool samples were collected at multiple time points: before pretreatment (week 0), after pretreatment prior to the first FMT (week 3), following each FMT (post-1 to post-4), and at weeks 8, 10, and 14. At week 14, a sigmoidoscopy was performed to assess clinical response (n = 9) and non-response (n = 15) (Supplementary Information S1).

### Microbiota data

DNA was extracted from donor and recipient stool samples and shotgun sequenced with 100 bp single-end reads to a median depth of 2.9 million reads by Diversigen (New Brighton, Minneapolis, USA) on Illumina NovaSeq platform. The mOTUs3 workflow (version 3.0.1) was used to generate taxonomic profiles (Supplementary Information S1), which resulted in 1552 unique mOTUs [18, 19]. For the sake of simplicity, we use the term “species” to refer to unique mOTUs throughout. The results table was then imported into R (version 4.2.2) and R code is available via GitHub (https://github.com/susannepinto/FECBUD_microbiome.git).

### Mapping ecological categories

Not all donor FMT samples were sequenced, complicating direct linkage of patient and donor samples. Therefore, we created a dataset with the core microbiota, species with relative abundance >0.1% in at least one sample, for each donor (n = 13 samples from D07 and n = 14 samples from D08). The core donor microbiota yielded 120 and 84 unique species for donors D07 and D08, respectively. Thresholds of 0.01%, 0.05%, 0.5%, and 1% were also tested in sensitivity analyses.

**Figure 1:**
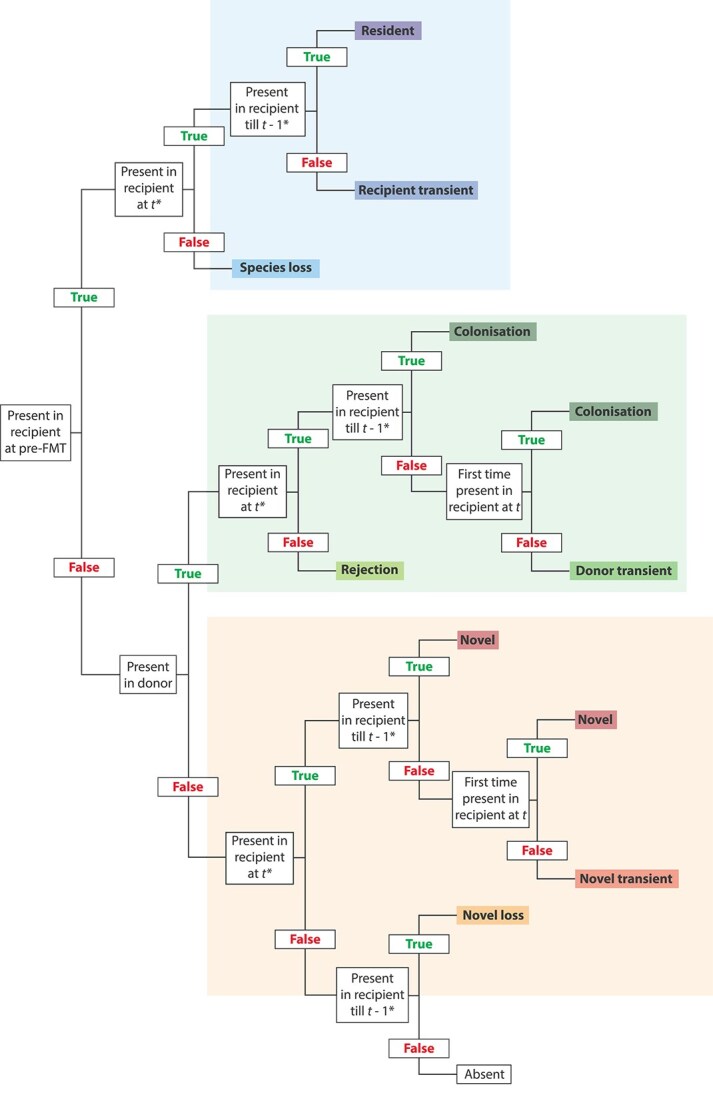
Decision tree used to assign species to ecological categories. The categories are based on the origin and presence of a species over time. First, the species was compared to the pre-FMT recipient samples, then to the core donor microbiota. Next, the presence/absence at all previous timepoints was considered to assign the species to an ecological category. Note that we ignored the first absence of species when categorising species as lost or as transient upon re-detection. In Sensitivity 1 we evaluated whether this choice had an impact on the results (details in Supplementary Information S2).

We examined the relative proportions of species unique to the recipient, unique to the donor, and shared between both. We created a presence/absence dataset of all species per recipient and per timepoint, and every species was assigned to an ecological category per recipient and per timepoint based on its origin and presence over time (decision tree in Fig. 1, details in Supplementary Information S2). Per recipient, for every species ever present at any timepoint in the recipient, or present in the microbiota of the associated donor, a comparison was made with the recipient’s pre-FMT sample and with the microbiota of the corresponding donor. All species present in the recipient’s pre-FMT sample were placed into a recipient category, with subcategories depending on the pattern of presence over time (Resident, Recipient transient, or Species loss). Species unique to the donor relative to the recipient’s pre-FMT samples were placed into a donor category (Colonisation, Donor transient, or Rejection). An additional sensitivity analysis categorised species shared between recipient and donor as donor species. Species absent in the recipient pre-FMT and donor were classified as a novel species (Novel, Novel transient, or Novel loss). Within the broad categories, a species was further categorised as a stable (Resident, Colonisation, or Novel), intermittent (Recipient transient, Donor transient or Novel transient), or previous occupant (Species loss, Rejection, or Novel loss) in the microbiota, depending on the presence at that moment and at previous timepoints. Because absence may reflect abundance below the detection limit, the base case allowed one single absence per species without affecting categorisation in the rest of the series. Due to the way the categories are defined, some categories cannot occur at the first timepoints. For example, a donor-derived species first had to colonise the gut (colonisation), then be absent for at least two timepoints (NA and Rejection), and then be detected again to be categorised as a Donor transient species (Supplementary Information S2). To provide a more comprehensive view, we also show the distribution of categorisation per family.

In sensitivity analyses, we tested variations to the base case criteria regarding the temporal information used for categorising species. In sensitivity categorisation approach 1 (Sensitivity 1) we did not allow any absence when categorising species into recipient, donor, or novel categories (Fig. 1). In sensitivity categorisation approach 2 (Sensitivity 2) we only considered the presence/absence at the previous timepoint instead of all previous timepoints (Supplementary Fig. S1). In contrast, in sensitivity categorisation approach 3 (Sensitivity 3) the presence of species at all timepoints is considered in categorisation of species at a particular timepoint (Supplementary Fig. S2). Sensitivity categorisation approach 4 (Sensitivity 4) is the same as Sensitivity 3 but with the added criterion of not allowing any absence (Supplementary Fig. S2). In Supplementary Information S2 examples on the differences between the sensitivity analyses are illustrated.

### Modelling the number of species across ecological categories

We modelled the number of species across ecological categories using overdispersed Poisson regression models with random effects to accommodate correlation between repeated measurements per recipient. We used a generalized linear mixed-effects model (GLMM) with a negative binomial family and a log-link using the “glmer.nb” function from the “lme4” R package [20]. The temporal evolution of the expected log-number of species in each category was modelled with a spline transformation of the original time variable (in weeks since start of FMT treatment). Estimates from the spline model were compared to those from a linear model in sensitivity analysis, by modelling the expected log-number of species as a simple linear function of time. Differences in succession dynamics between responders and non-responders were investigated by adding the treatment response variable as a covariate to the model, and through specification of interaction terms with time and ecological category. The interaction determined whether there was a divergence in the numbers in a particular category between non-responders and responders, with statistical significance assessed by Wald tests. Patient specific variables, donor (donor D07 vs. D08), pretreatment (budesonide vs. placebo), age and sex (female vs. male), were included based upon their role as possible confounders.

### Change in species population abundances

To explore the dynamics of species in response to FMT in more detail, we investigated the relative abundance over time for the species present in the recipient pre-FMT, donor species, and novel species. In addition, we compared the baseline distributions among species that were later categorised as Resident, Recipient transient, and Species loss among both responders and non-responders. Finally, we also calculated the differences in microbial abundance before and after FMT for all species that were present in the recipients’ pre-FMT samples. Because several non-responder patients quitted early, we only included patients who completed all four rounds of FMT and had at least one post-FMT sample (n = 18 patients, of whom nine were defined as responders) and used the last available post-FMT measurement when calculating the difference in relative abundance before and after FMT. Because the abundance distributions were right-skewed, we used a natural log transformation of the abundances. Consequently, the abundance differences on the log scale can be interpreted as proportional differences on the original scale (in percentage difference). Significance was assessed by linear mixed-effects models, accounting for repeated observations within each patient (using the “lmer” function from the “lme4” R package) [20].

## Results

### Distribution of shared and unique donor and recipient species

First, we examined the relative proportions of species unique to the recipient, unique to the donor, and shared between donors and their corresponding recipients. This analysis also informs our classification approach, as shared species were categorised as recipient species in base case analyses. The proportion of unique recipient species fluctuated around 50%, with a tendency toward higher proportions (Supplementary Fig. S3). Notably, responders appeared to have a higher proportion of species unique to the recipient compared to non-responders, though some exceptions were observed, e.g. patient 102 exhibited a high proportion of unique recipient species despite being a non-responder. The proportion of shared species was relatively small compared to unique core donor species (Supplementary Fig. S3). However, when these shared species were categorised as recipient species, they made up a considerable fraction of the recipient-associated microbiota in our classification.

### Succession of recipient-derived, donor-derived, and novel species following FMT

**Figure 2:**
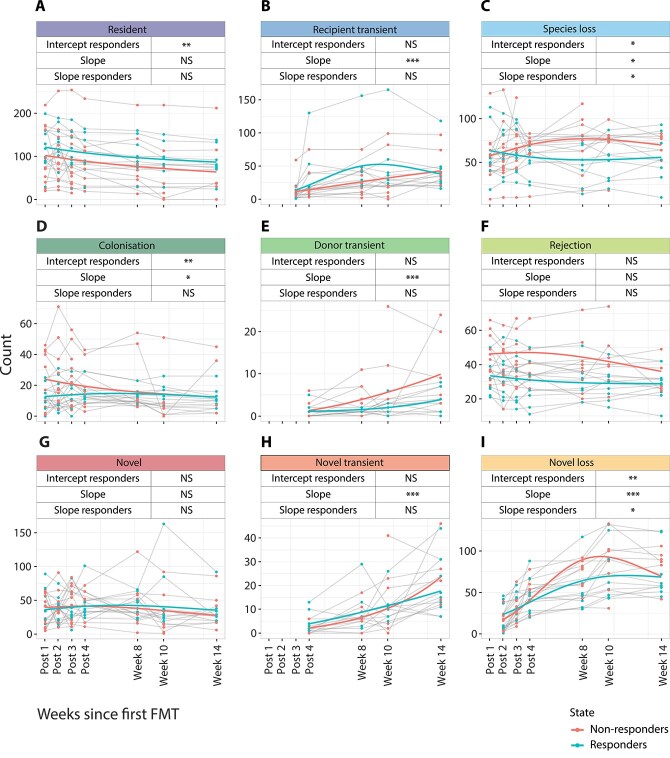
Changes in the number of species per ecological category over time. The term "intercept responders" captures differences in the baseline numbers of species at the start of treatment between responders and non-responders. The term "slope" represents the overall trajectory of change in species numbers over time. The term "slope responders" indicates whether the rate of change over time differed between responders and non-responders. Average trajectories and individual patient trajectories are shown. Note the different scaling of the y-axis per category. The model contained a random intercept per patient to account for within-patient correlation due to repeated measurements. Time was modelled with a spline. Statistical significance was assessed using Wald tests. The levels of significance are reported above each plot and are indicated by asterisks (^***^ = *P* < .01; ^**^ = *P* < .01; ^*^ = *P* < .05; NS = not significant).

To study the succession dynamics of species during and after FMT in our UC cohort, we modelled the number of species across ecological categories and investigated differences between responders and non-responders (Fig. 2). In these models, donor and sex were included as covariates, while pretreatment and age were excluded as confounders. Supplementary Fig. S4 shows the specific parameter estimates of the model depicted in Fig. 2.

At the start of the study, we observed a significantly higher number of recipient species in the resident categories (species that were present in the patient’s gut pre-FMT) among responders compared to non-responders, and this difference persisted over time (Fig. 2A). Although the number of resident species declined over time in both responders and non-responders, this decrease was not statistically significant. In contrast, the number of recipient transient species increased significantly over time in both patient groups (Fig. 2B). Of note, this increase may be partly attributable to the definition of recipient-derived species being transient upon re-detection after temporary absence. Non-responder patients exhibited a significantly greater loss of recipient species over time compared to responders, in whom the number of recipient species lost decreased significantly over time (Fig. 2C).

Conversely, non-responders were initially colonised by a significantly higher number of donor species compared to responders. However, the number of colonising species in non-responders declined over time, whereas it remained constant in responders (Fig. 2D). The number of donor transient species was similar between the two patient groups at the start of the study and showed a significant increase over time, especially in non-responders. However, this category remained relatively small and differences according to treatment response were not significant (Fig. 2E). The number of rejected donor species was higher at baseline and over time for non-responders compared to responders, however this difference also did not reach statistical significance (Fig. 2F).

The number of novel species detected post-FMT was similar for both responders and non-responders and remained constant in time (Fig. 2G). The number of novel transient species increased significantly over time, this increase was similar for both the responders and non-responders (Fig. 2H). Initially, the responders lost significantly more novel species than the non-responders, but over time the latter group lost significantly more novel species than the responders (Fig. 2, panel I).

We also found significant differences between responders and non-responders in the recipient transient and novel transient categories when applying a linear model instead of splines for the temporal evolution of number of species in each category (Supplementary Fig. S5). It should be noted that these categories contained relatively few species, and the lack of statistical significance when using splines is likely explained by a reduced statistical power. Importantly, all differences between responders and non-responders identified by the spline model were retained in the linear model for number of species in each category (Supplementary Fig. S5).

When examining microbial families per category, we observed that the families with the highest species diversity had representatives in all categories (Supplementary Fig. S6). Prevotellaceae was of particular interest, as previous research highlighted its importance in distinguishing responders from non-responders [11]. However, even within this family, species were distributed across multiple categories. Some families, such as Campylobacteraceae, Dehalococcoidales fam. *i.s.*, and Flavobacteriaceae, were found exclusively in novel categories, though their species counts were relatively low. Additionally, while some families were assigned solely to recipient categories (e.g. Bacillales fam. *i.s.* and Mollicutes fam. *i.s.*), no families were restricted to donor categories (Supplementary Fig. S6).

### Sensitivity analyses

Choosing a smaller threshold (0.01% or 0.05%) compared to the base case did not notably alter the results, indicating that the classification of species as core donor microbiota was stable across these thresholds (Supplementary Fig. S7). However, the larger thresholds (0.5% and 1%) resulted in a shrinkage to zero of the donor species' estimates in Poisson regression. This shift suggests that fewer donor species were classified at these higher thresholds.

When shared species were categorised as donor species rather than resident species, some previously significant differences were no longer statistically significant (Supplementary Fig. S8). Under this alternative classification, non-responders still exhibited a greater loss of recipient species over time compared to responders. However, while the loss of recipient species in responders appeared to decline over time, this difference was no longer significant. Similarly, the number of colonising species remained lower in responders than in non-responders, but this difference was no longer statistically significant. In contrast, while our initial classification suggested a smaller increase in donor transient species over time for responders, without a significant difference from non-responders, this effect now became more pronounced and statistically significant. Lastly, in our original classification, responders lost significantly more novel species than non-responders. This trend persisted under the revised classification but was no longer statistically significant (Supplementary Fig. S8).

We conducted four different sensitivity analyses concerning the categorisation of the species. To illustrate the effect of categorisation on the rates of change over time, we generated a plot of the average slope estimates according to each sensitivity analysis (Supplementary Figs. S9-S13). Sensitivity categorisation approach 1 resulted in a slightly stronger decline in the number of species for the resident, colonisation, and novel categories (Supplementary Figs. S9, S13, and S14). This outcome is a logical consequence of the criterion that a species can no longer be absent for a single time point. Consequently, the likelihood of a species moving to a different category (transient or loss) increased, since it was by definition not possible to return to the categories denoting stable presence over time. This resulted in transient categories having higher intercepts, but the average slopes remained unchanged for all other categories (Supplementary Figs. S9, S13, and S14). Similarly, for Sensitivity categorisation approach 2, no substantial differences from the base case were found (Supplementary Figs. S10, S13, and S14). The most profound differences were noted in the slopes of the resident and transient categories. The slopes of the transient categories were smaller, especially for the recipient-derived species among non-responders (Supplementary Figs. S10, S13, and S14). Sensitivity categorisation approaches 3 and 4 led to more stable patterns over time, especially for the resident category, as compared to both the base case scenario and the other sensitivity analyses (Supplementary Figs. S11–S14). This stability can be attributed to the modifications in the category assignment criteria in Sensitivity categorisation approaches 3 and 4, where stable presence is defined on all timepoints. Consequently, fewer species were assigned to the resident, colonisation and novel categories and more to the transient categories (Supplementary Information S2).

### Relative abundances of species pre- and post-FMT

**Figure 3:**
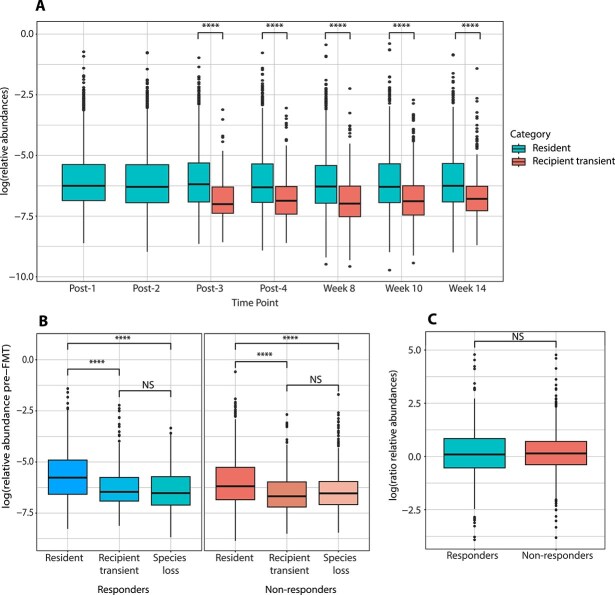
Comparison of relative abundances of recipient species in different categories. (A) Relative abundances of resident and recipient transient species over time. Here, no distinction has been made between responders and non-responders. (B) Relative abundance of recipient species at pre-FMT measurement. The relative abundances in species categorised as resident, recipient transient, and species loss between responders and non-responders are not significant (Supplementary Table S2). (C) Difference in relative abundance in resident species between pre-FMT and last available post-FMT measurement for responders and non-responders. Significance was tested with linear mixed-models and shown in the plots (^***^ = *P* < .01; ^**^ = *P* < .01; ^*^ = *P* < .05; NS = not significant).

We further assessed changes in the relative abundance of species per category and investigated whether the relative abundance pre-FMT is indicative of the category that a species will reach post-FMT. Recipient transient species displayed significantly lower relative abundances at all timepoints compared to resident species (Fig. 3A and Supplementary Table S2). Donor species exhibited a similar pattern, with donor transient species generally showing lower relative abundance than colonising species. However, this difference was less pronounced than what we observed for recipient species and was not statistically significant at all time points (Supplementary Fig. S15A). Novel species did not display a clear distinction between transient and stable groups, and the overall differences in their abundance were also less pronounced than for recipient species. However, novel transient species tended to exhibit fewer extreme outliers with high abundances compared to novel species, and in some cases, this difference reached statistical significance (Supplementary Fig. S15B). Our next analysis revealed that, across most time points, resident species exhibit significantly higher abundances compared to colonising and novel species, with novel species showing the lowest abundances overall (Supplementary Fig. S16).

In both responders and non-responders, recipient species with higher pre-FMT relative abundances were more likely to remain in the recipient’s gut and become resident species, compared to recipient species that were transient or lost (Fig. 3B, Supplementary Fig. S17, and Supplementary Table S2). Therefore, our findings show that initial microbiota composition is associated with post-FMT composition. The differences in relative abundance of recipient resident species between the pre-FMT measurement and the last available post-FMT measurement were centered around zero (Fig. 3C). A positive difference indicates an increase in the relative abundance of resident species following FMT, while a negative difference denotes a decrease. Thus, approximately equal numbers of resident species showed either a positive or negative response to FMT. No significant differences were found between responders and non-responders in relative abundances of resident species in response to FMT (Fig. 3C, Supplementary Fig. S18, and Supplementary Table S2).

## Discussion

The success of FMT for UC is determined by whether the patient achieves clinical and endoscopic remission after treatment. The influence of donor microbiota characteristics on the efficacy of FMT remains a topic of debate. It has been suggested that treatment success is related to how much the recipient’s microbiota composition shifts towards that of the donor [7, 21]. However, we found no evidence supporting this link, in line with several other studies [3, 10–13, 16, 22]. Instead, our longitudinal analysis of an FMT trial, involving 22 UC patients treated with four rounds of FMT donated by two healthy donors, suggests complementary contributions of donor and recipient species in shaping clinical response. In our earlier study, we observed differences between the microbiota of donors and recipients post-FMT. Among patients who responded well to treatment, gut microbiota composition did not fully transition to resemble that of the donor, suggesting that recipient factors also play a role in microbiota reconstitution [11]. Furthermore, we found that engraftment was not predictive of achieving remission in UC [16], consistent with previous reports emphasizing the role of recipient microbiota composition and dysbiosis in shaping post-FMT outcomes [12, 22].

In previous publications of this dataset, we observed significant differences in treatment efficacy between donors, with one donor associated with a superior response compared to the other [11, 16]. Similarly, a previous study also reported a donor-dependent effect of FMT in UC patients [3]. However, our findings do not support the "super-donor" hypothesis, which posits that specific donor microbiome features are the primary determinants of FMT success [7]. Rather than colonisation by donor species, recipient factors, such as microbial richness and compositional divergence from healthy donors may outweigh donor factors in driving FMT clinical outcomes [12]. Our previous *α*-diversity analysis indeed showed that responders had a lower Simpson dominance (i.e. a higher diversity) than non-responders [11]. Additionally, *β*-diversity analyses, using Aitchison distance, demonstrated that patients whose microbiota became more donor-like over time were more often non-responders [11].

In this study, we used an ecological framework of succession to investigate microbiota dynamics associated with clinical success of FMT. Microbial species were categorised as pre-existing in the recipient before FMT, donor-derived, or newly detected. Our study expands upon previous analyses by using longitudinal analysis of UC patients, thereby providing a fine-grained view of the ecological dynamics over time of donor and recipient species following FMT. We found that responders retained a higher number of recipient species compared to non-responders. Although non-responders initially exhibited colonisation by more donor species than responders, this colonisation in non-responders declined over time and eventually became equal to the levels observed in responders. These findings suggest that a successful clinical response to FMT may be facilitated by a microbiota receptive to colonisation without compromising the resident microbiota. Additionally, non-responders lost substantially more novel species over time compared to responders, indicating that newly detected species failed to establish stably within the non-responder gut microbiota. This suggests less robust alterations in gut microbiota composition among non-responders. A successful FMT may induce a shift in which the recipient’s microbiota integrates donor and novel species, achieving a balanced coexistence to restore the gut microbial ecosystem. This aligns with earlier research [12, 23].

The success of FMT may not be reliant on resembling the donor’s microbiota, but rather on establishing a complementary relationship, emphasizing the importance of selecting donors whose microbiota aligns with the recipient’s specific needs [7]. However, the pre-existing microbiota makes the introduction of new species and the induction of change considerably more challenging [24, 25]. FMT can be seen as a perturbation experiment on the gut microbiota, creating a dynamic interplay between donor and recipient communities [12, 26]. This post-FMT community arises due to a combination of ecological processes and stochastic factors such as niche availability, competitive exclusion, and environmental influences [12, 27]. The stability of the microbiota is maintained through controlled species loss, ensuring that introduced organisms integrate harmoniously with the pre-existing ecosystem. Importantly, the properties of this newly established community can be either beneficial or dysbiotic, depending on the specific composition and functional roles of the new community and its compatibility with the recipient. The balance between the colonisation of beneficial microorganisms and competition with deleterious microorganisms in the recipient gut, combined with systemic recipient processes, such as the modulation of immune responses, increased diversity, and the interaction with (external) environmental factors and genetic characteristics, could initiate clinical remission [4]. The complexity of this balance may also explain why prolonged FMT treatment with multiple donor infusions appears necessary in UC, as repeated exposition may be required to achieve a functional balance between recipient and donor microbiota. This approach contrasts with the FMT treatment of recurrent *Clostridioides difficile* infections, which is characterized by a depleted microbiota that can be effectively restored with a single infusion, with a cure rate of ~80% [1]. High gut microbial diversity in the donor and low diversity (dysbiosis) in the recipient may influence the success of colonisation [10, 28].

We observed that species with a higher abundance prior to FMT are more likely to persist during the FMT than species with a lower abundance. This implies that the competitive strength of the resident species is related to their abundance, indicating that within each metabolic niche, communities are built by random winners, driven by stochastic colonisation [29]. This aligns with the expectation that newly introduced species may not always establish successfully even if they are introduced in high abundance, while resident species are likely to first decline in abundance before being lost entirely [3, 22]. Previous ecological studies showed that functional differences create opportunities for coexistence (niche theory). However, within each niche functionally similar species can coexist, and communities are structured to random stochastic rules (neutral theory) [30]. Within the gut microbiota, species often have overlapping functions, allowing them to replace each other and take over specific functional traits if one species is perturbed or removed [31].

This study has several limitations. The first concerns the classification of patients into responders and non-responders. Patients that dropped out early due to worsening symptoms were classified as non-responders. Microbiota data were not collected for these patients, which may bias the results for the non-responder group. Moreover, the study concerns only 22 UC patients and the time series up to 14 weeks represents only a short window of the dynamic process of microbial succession. Further investigation into longer-term outcomes is necessary to gain a more comprehensive understanding [32]. Therefore, we encourage other researchers to create larger longitudinal datasets and utilize the methods presented here to further investigate FMT in the context of microbial succession. A third limitation is the sequencing depth (2.9 million 100 bp single-end Illumina reads), which does not allow for definitive determination of whether an absent species was actually absent in the recipient or donor, or simply undetected [33]. As a result, some initially low-abundance resident species may have been mistaken for donor-derived or novel species, and some low-abundance donor-derived species may have been misclassified as novel. Furthermore, the low sequencing resolution prevented within-species genome comparisons, which would be necessary to distinguish between strain transfer or strain coexistence. Our study captures microbial dynamics on the species rather than the strain level; by not accounting for possible displacement of resident by donor-derived strains, we may have underestimated the degree of successful colonisation after FMT. However, regardless of the precise origin, the continued presence or emergence of these species to detectable levels following FMT suggests that transfer of the donor material induced favorable ecological conditions that facilitated their proliferation or contributed to their ecological stability. Whether they are significantly contributing to the overall functionality, and could impact the clinical outcome for the recipient, remains to be investigated. Fourth, we recognize that we cannot fully determine if the observed changes in recipient species’ absence would have occurred naturally over time, independent of FMT intervention. Furthermore, we did not have data to directly link the unique donor sample used for FMT to the corresponding recipient samples. Therefore, we used the donor core microbiota data. However, a considerable period had elapsed between these samples (~1 year and 9 months for Donor D07 and 1 year and 5 months for Donor D08), during which host-related factors, such as dietary changes or travel, may have influenced the donor microbiota composition [16]. As previously shown, even family-level composition varied slightly between timepoints [11]. This temporal variability is not captured when using a core microbiota approach, and may have led to the misclassification of some low abundant colonising species from the donor as novel species. Nonetheless, all FMTs for a given patient were derived from donor samples collected within the same general period, minimizing the potential for intra-treatment variability. Moreover, sensitivity analyses on the threshold used to define the core microbiota showed that the set of core taxa was relatively robust to moderate changes in threshold. Lastly, while Donor D08 showed relatively higher success in transfers, the sample size was insufficient to perform per donor analyses. Future research should aim to include a more diverse cohort of donors, to investigate donor-specific variations.

Our study is exploratory and does not offer definitive evidence, as conclusions are affected by the small sample size and limited depth and resolution of the sequencing technique used. Rather, we aimed to introduce a new conceptual framework rooted in ecological theory that can contribute to a better understanding of microbiome dynamics. By applying an ecological perspective to FMT, our study sheds new light on the importance of ecological principles, such as succession of microorganisms and the resilience of the recipient’s system, in shaping therapeutic outcomes. Our study reveals the ecological dynamics of the gut microbiota during and after FMT in patients with UC, with a particular focus on the dynamics of recipient, donor, and novel species. Contrary to some previous studies, the rate of colonisation by species from the donor microbiota did not emerge as a beneficial factor for FMT success in this study [7, 13]. Rather, clinical response was associated with the recipient’s ability to retain resident species while also incorporating novel and donor species. Thus, successful FMT hinges on fostering a microbiota shift that complements rather than compromises the existing ecosystem. This ecological interpretation aids in understanding the mechanism through which FMT may induce clinical remission and also underscores the nuanced interplay between donor and recipient microbiota essential for therapeutic efficacy.

## Supplementary Material

Supplementary_Figure_S1_ycaf119

Supplementary_Figure_S2_ycaf119

Supplementary_Figure_S3_ycaf119

Supplementary_Figure_S4_ycaf119

Supplementary_Figure_S5_ycaf119

Supplementary_Figure_S6_ycaf119

Supplementary_Figure_S7_ycaf119

Supplementary_Figure_S8_ycaf119

Supplementary_Figure_S9_ycaf119

Supplementary_Figure_S10_ycaf119

Supplementary_Figure_S11_ycaf119

Supplementary_Figure_S12_ycaf119

Supplementary_Figure_S13_ycaf119

Supplementary_Figure_S14_ycaf119

Supplementary_Figure_S15_ycaf119

Supplementary_Figure_S16_ycaf119

Supplementary_Figure_S17_ycaf119

Supplementary_Figure_S18_ycaf119

Supplementary_Table_S1_ycaf119

Supplementary_Table_S2_ycaf119

Supplementary_Information_S1_ycaf119

Supplementary_Information_S2_ycaf119

## Data Availability

R code is available via GitHub (https://github.com/susannepinto/FECBUD_microbiome.git) and the in-house preprocessing workflow is available via https://git.lumc.nl/snooij/metagenomics-preprocessing. We have uploaded the metagenomic sequences to NCBI with: SRA Bioproject PRJNA1071720.
