# Supplementary material for: Ecological resilience in ulcerative colitis: microbial dynamics of donor and resident species in a longitudinal fecal microbiota transplantation study
Source: ISME Commun. 2025 Jul 16;5(1):ycaf119. doi: 10.1093/ismeco/ycaf119 (PMC12378841; doi:10.1093/ismeco/ycaf119)
Supplement: Supplementary_Figure_S1_ycaf119 [file supplementary_figure_s1_ycaf119.pdf]

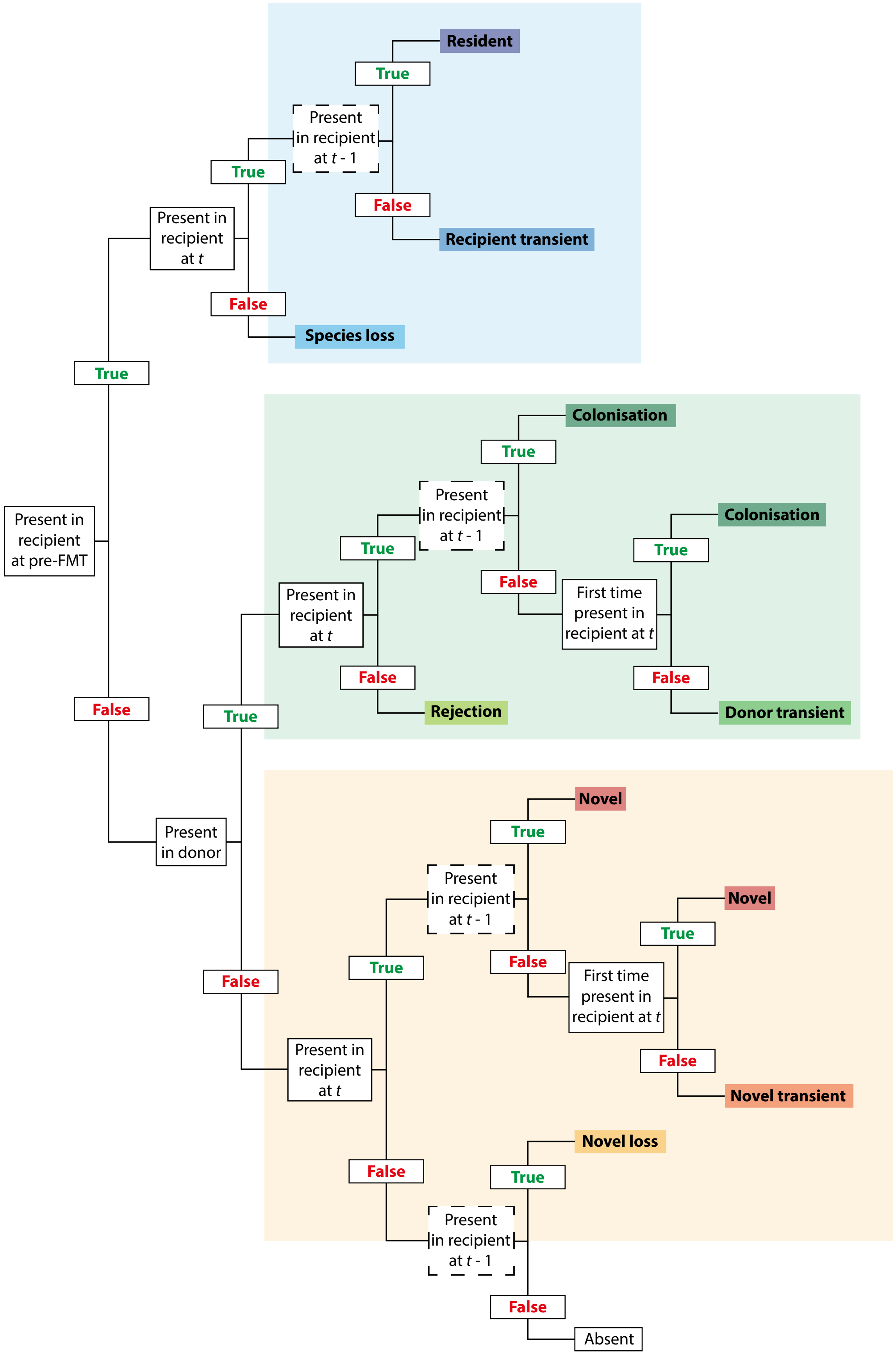

Supplementary Figure S1. Decision tree for Sensitivity 2 analysis to assign species to ecological categories according to different inclusion criteria as in the base case analysis. *The categories are based on the origin and presence of a species over time. First, the species was compared to the pre-FMT recipient samples, then to the core donor microbiota. Next, the presence/absence at only the previous timepoint was considered to assign the species to an ecological category. Differences with the base case scenario, where all previous timepoints were considered, are indicated with a dotted line around the box (see also Supplementary Information S2).*
