# Supplementary material for: Ecological resilience in ulcerative colitis: microbial dynamics of donor and resident species in a longitudinal fecal microbiota transplantation study
Source: ISME Commun. 2025 Jul 16;5(1):ycaf119. doi: 10.1093/ismeco/ycaf119 (PMC12378841; doi:10.1093/ismeco/ycaf119)
Supplement: Supplementary_Figure_S2_ycaf119 [file supplementary_figure_s2_ycaf119.pdf]

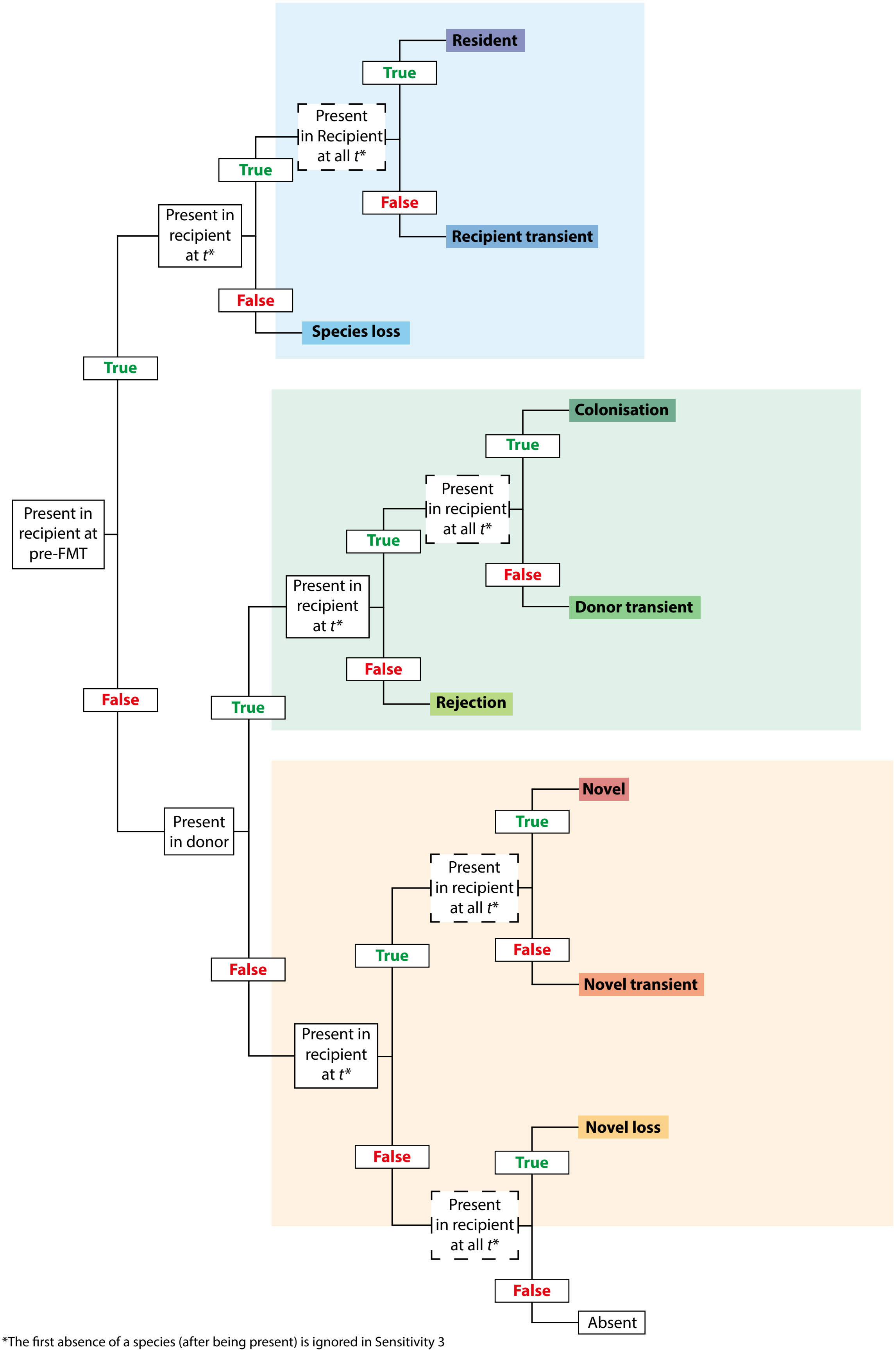

*The first absence of a species (after being present) is ignored in Sensitivity 3

Supplementary Figure S2. Decision tree for Sensitivity 3 and 4 analyses to assign species to ecological categories according to different criteria as in the base case analysis. *The categories are based on the origin and presence of a species over time. First, the species was compared to the pre-FMT recipient samples, then to the core donor microbiota. Next, the presence/absence at all timepoints was considered to assign the species to an ecological category. Differences with the base case scenario, where only previous timepoints were considered, are indicated with a dotted line around the box (see also Supplementary Information S2).*
