# Supplementary material for: Ecological resilience in ulcerative colitis: microbial dynamics of donor and resident species in a longitudinal fecal microbiota transplantation study
Source: ISME Commun. 2025 Jul 16;5(1):ycaf119. doi: 10.1093/ismeco/ycaf119 (PMC12378841; doi:10.1093/ismeco/ycaf119)
Supplement: Supplementary_Figure_S3_ycaf119 [file supplementary_figure_s3_ycaf119.pdf]

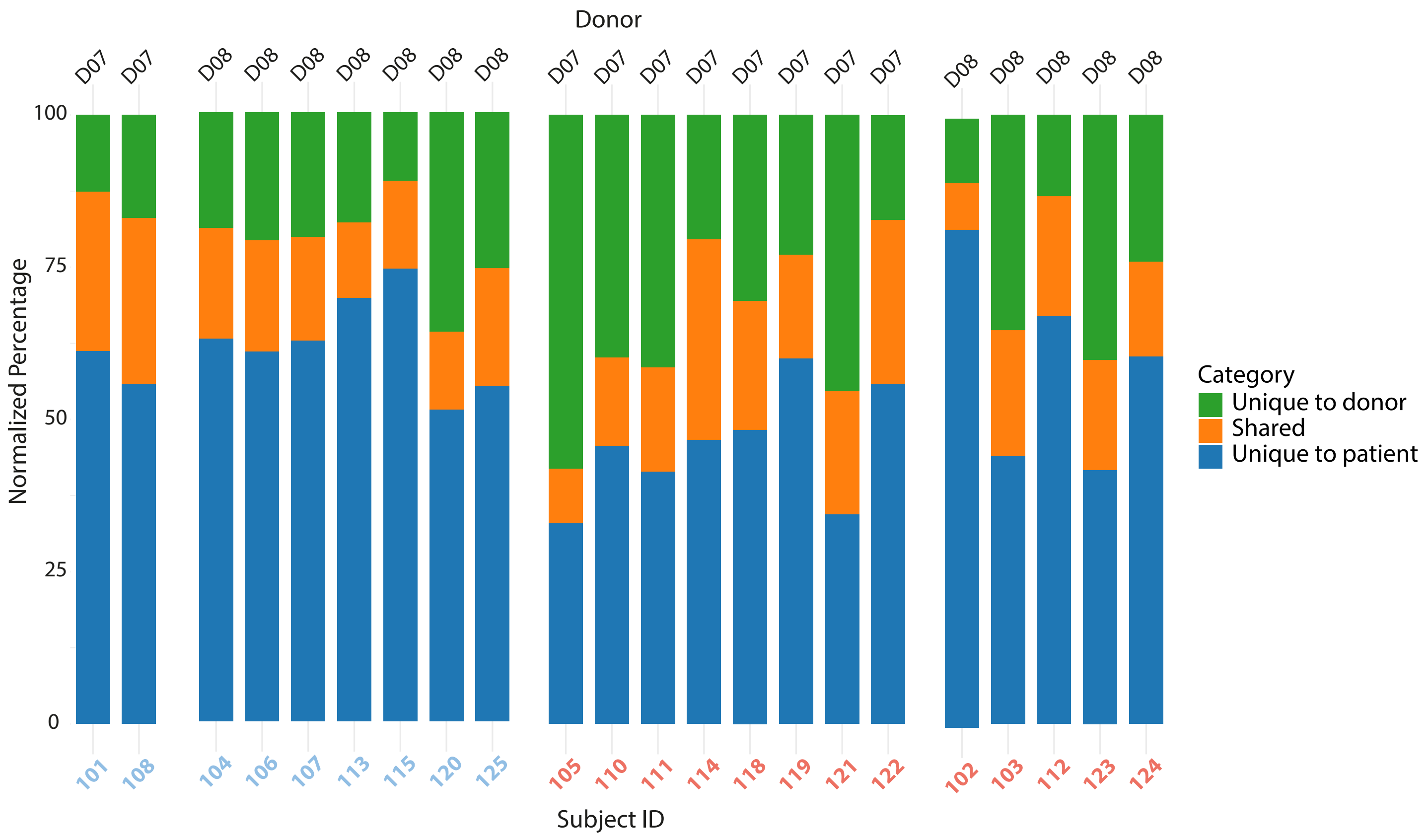

Supplementary Figure S3. Proportions of unique and shared species pre-FMT in FMT recipients. *Bar plots show the distribution of recipient species (mOTUs) for each patient. Bars represent the percentage of species categorised as Unique to patient (bottom), Shared with donor (middle), and Unique to donor (top). Upper x-axis gives the related donor, lower x-axis gives the responders (left-hand side) and non-responders (right-hand side).*
