# Supplementary material for: Ecological resilience in ulcerative colitis: microbial dynamics of donor and resident species in a longitudinal fecal microbiota transplantation study
Source: ISME Commun. 2025 Jul 16;5(1):ycaf119. doi: 10.1093/ismeco/ycaf119 (PMC12378841; doi:10.1093/ismeco/ycaf119)
Supplement: Supplementary_Figure_S4_ycaf119 [file supplementary_figure_s4_ycaf119.pdf]

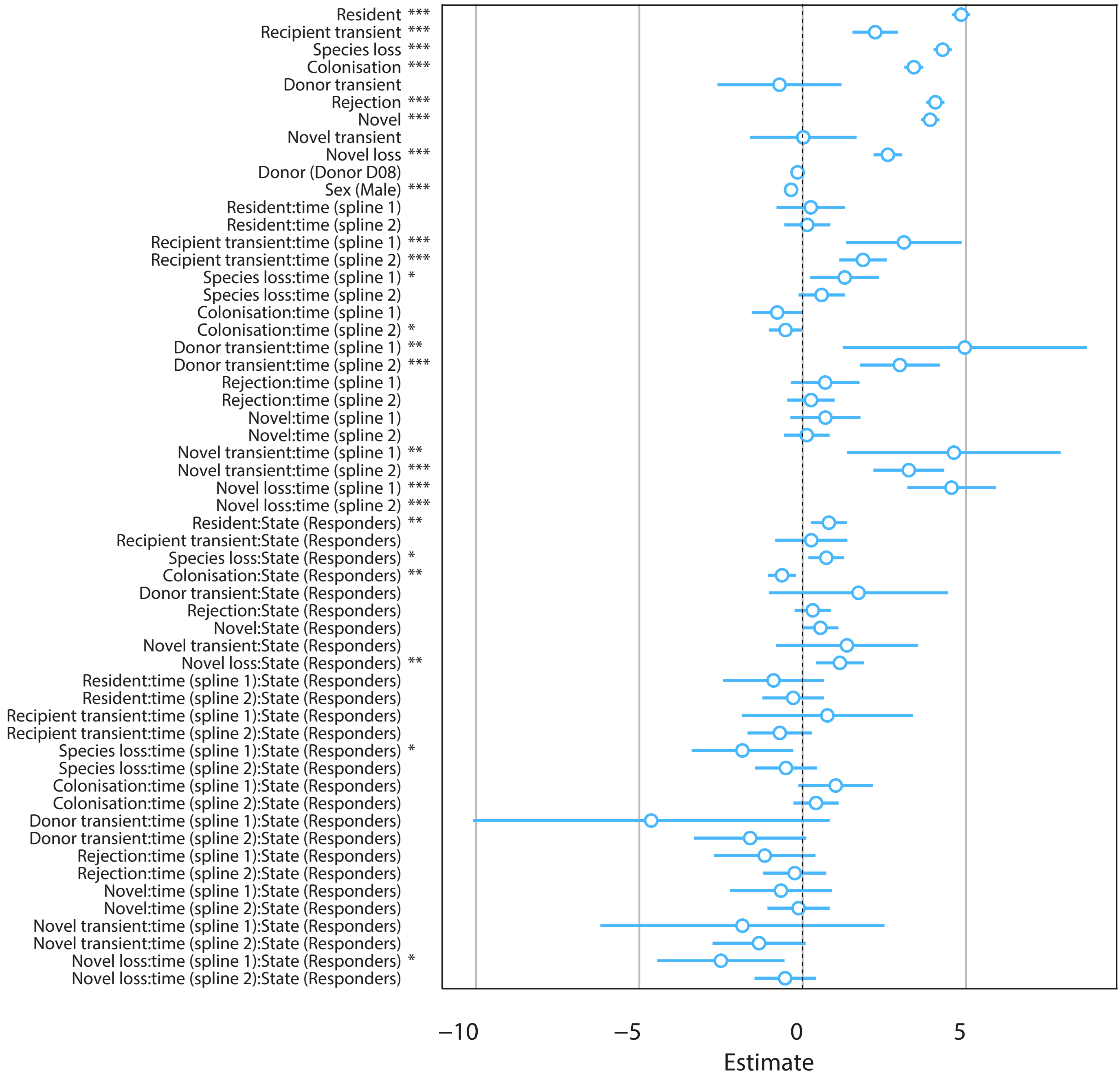

Supplementary Figure S4. Results of modelling (with a spline) the number of species per ecological category in the base case. *The point estimates, 95% confidence intervals, and a reference line at 0 are shown. When the horizontal lines do not cross the vertical reference line, this means that the coefficients are significantly different from 0. The original time variable was modelled with a spline rescaled to denote time in weeks since start of FMT. The model contained a random intercept per patient to account for repeated measurements.*
