# Supplementary material for: Ecological resilience in ulcerative colitis: microbial dynamics of donor and resident species in a longitudinal fecal microbiota transplantation study
Source: ISME Commun. 2025 Jul 16;5(1):ycaf119. doi: 10.1093/ismeco/ycaf119 (PMC12378841; doi:10.1093/ismeco/ycaf119)
Supplement: Supplementary_Figure_S5_ycaf119 [file supplementary_figure_s5_ycaf119.pdf]

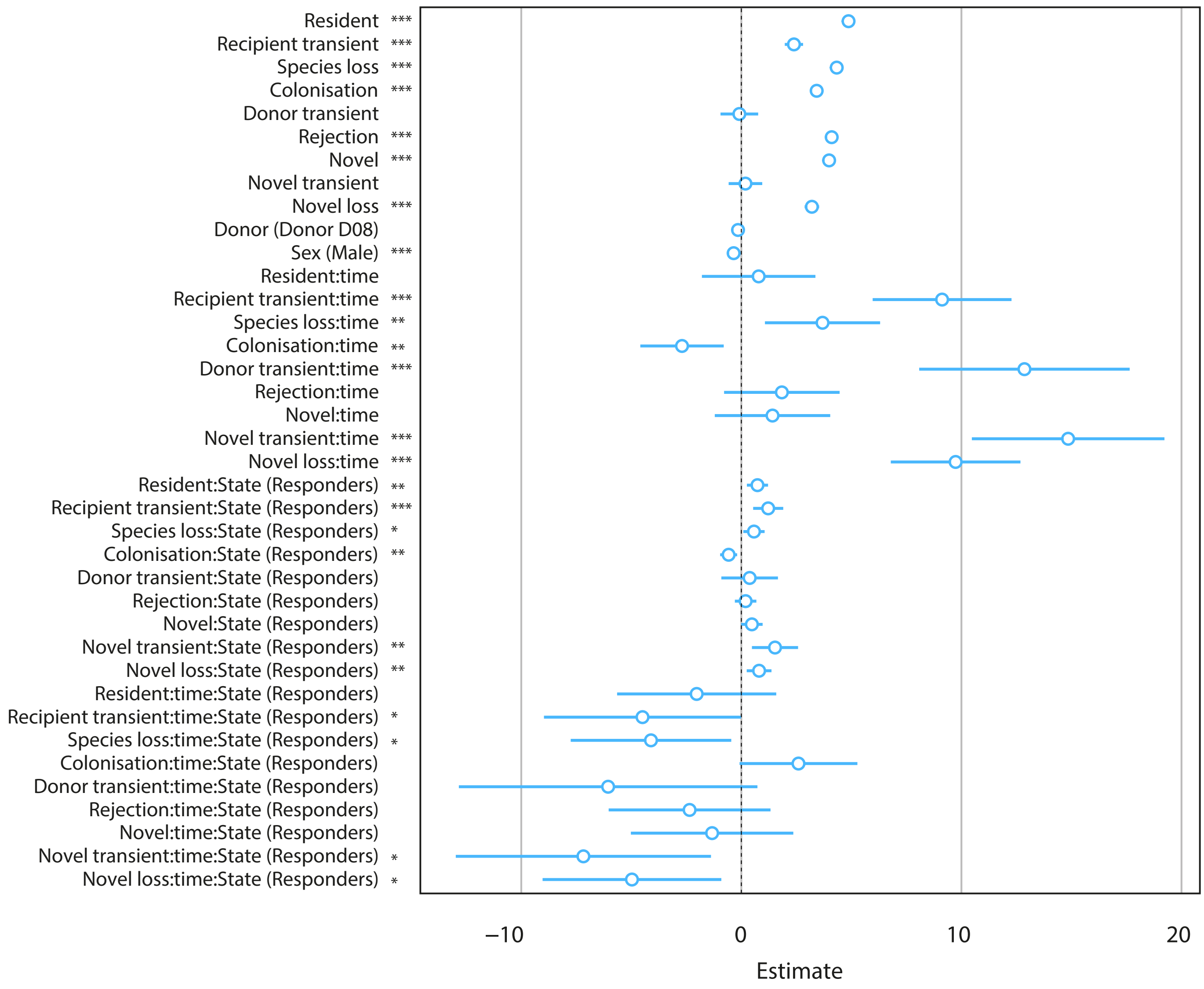

Supplementary Figure S5. Results of modelling (without a spline) the number of species per ecological category in the base case. *The point estimates, 95% confidence intervals, and a reference line at 0 are shown. When the horizontal lines do not cross the vertical reference line, this means that the coefficients are significantly different from 0. Contrary to the base case, the original time variable was not modelled with a spline. Time was rescaled to denote time in weeks since start of FMT. The model contained a random intercept per patient to account for repeated measurements.*
