# Supplementary material for: Ecological resilience in ulcerative colitis: microbial dynamics of donor and resident species in a longitudinal fecal microbiota transplantation study
Source: ISME Commun. 2025 Jul 16;5(1):ycaf119. doi: 10.1093/ismeco/ycaf119 (PMC12378841; doi:10.1093/ismeco/ycaf119)
Supplement: Supplementary_Figure_S6_ycaf119 [file supplementary_figure_s6_ycaf119.pdf]

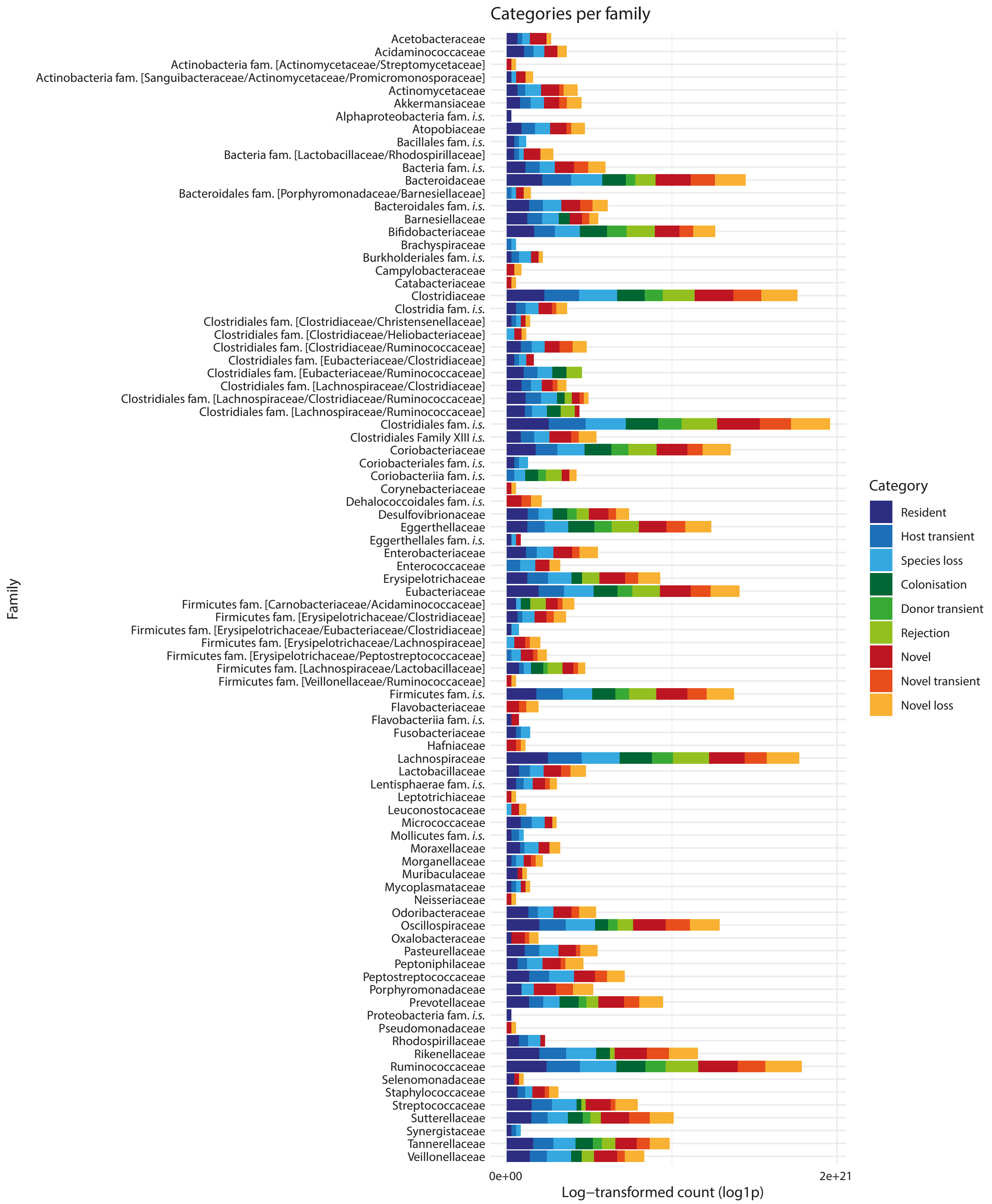

Supplementary Figure S6. Families per ecological category in the base case. *The x-axis shows the log-transformed counts of species per family, the colors indicate in which categories the species were placed. Note that categories can change over time, but this is not shown here.*
