# Supplementary material for: Ecological resilience in ulcerative colitis: microbial dynamics of donor and resident species in a longitudinal fecal microbiota transplantation study
Source: ISME Commun. 2025 Jul 16;5(1):ycaf119. doi: 10.1093/ismeco/ycaf119 (PMC12378841; doi:10.1093/ismeco/ycaf119)
Supplement: Supplementary_Figure_S11_ycaf119 [file supplementary_figure_s11_ycaf119.pdf]

Sensitivity 3

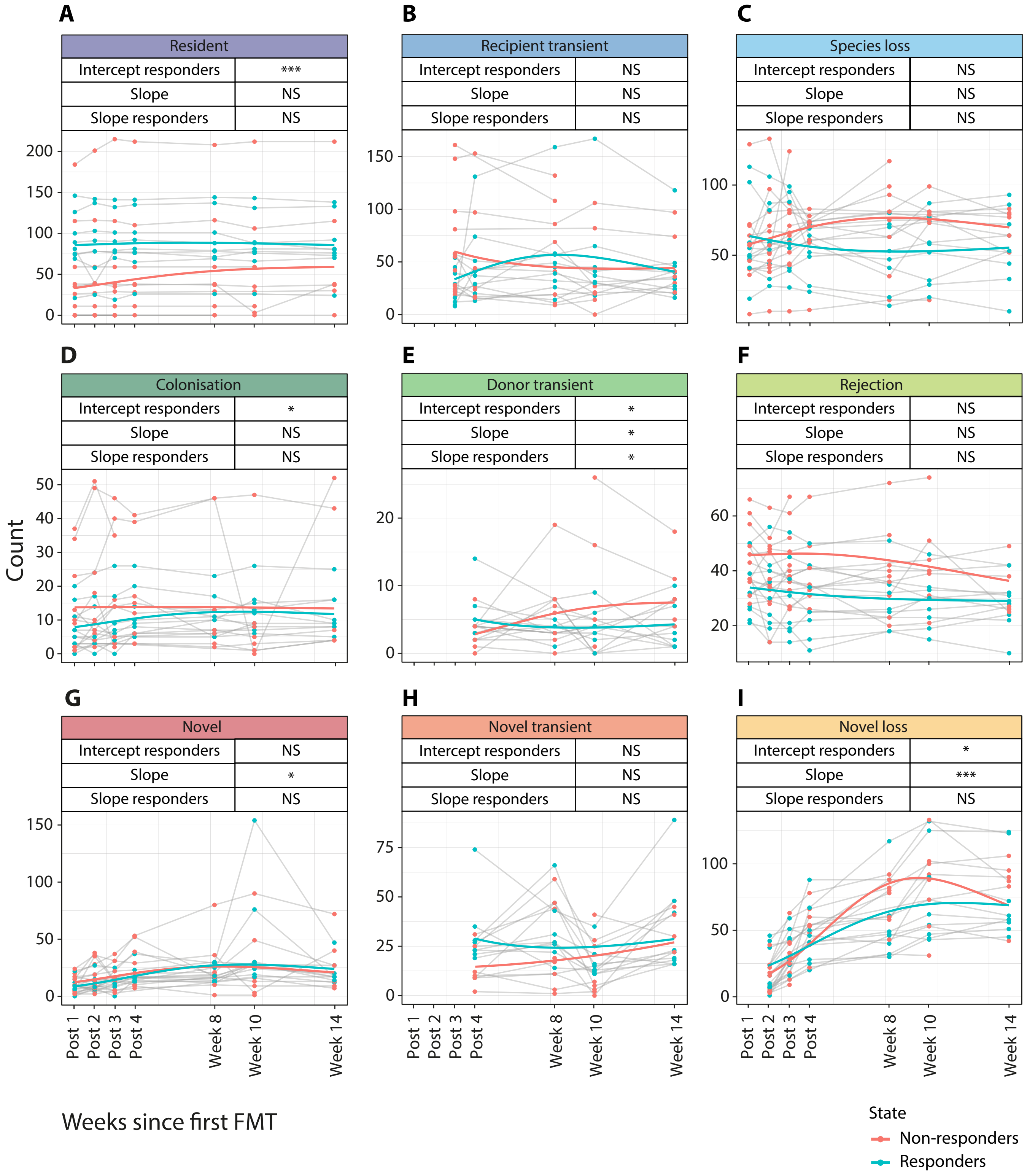

Supplementary Figure S11. Temporal changes in the number of species per ecological category for Sensitivity 3. *The term "intercept responders" captures differences in the baseline numbers of species at the start of treatment between responders and non-responders. The term "slope" represents the overall trajectory of change in species numbers over time. The term "slope responders" indicates whether the rate of change over time differed between responders and non-responders. Average trajectories and individual patient trajectories are shown. Note the different scaling of the y-axes. The model contained a random intercept per patient to account for repeated measurements. Time was modelled with a spline. The levels of significance are reported above each plot and are indicated by asterisks (*** = p-value < 0.01; ** = p-value < 0.01; * = p-value < 0.05; NS = not significant).*
