# Supplementary material for: Ecological resilience in ulcerative colitis: microbial dynamics of donor and resident species in a longitudinal fecal microbiota transplantation study
Source: ISME Commun. 2025 Jul 16;5(1):ycaf119. doi: 10.1093/ismeco/ycaf119 (PMC12378841; doi:10.1093/ismeco/ycaf119)
Supplement: Supplementary_Figure_S14_ycaf119 [file supplementary_figure_s14_ycaf119.pdf]

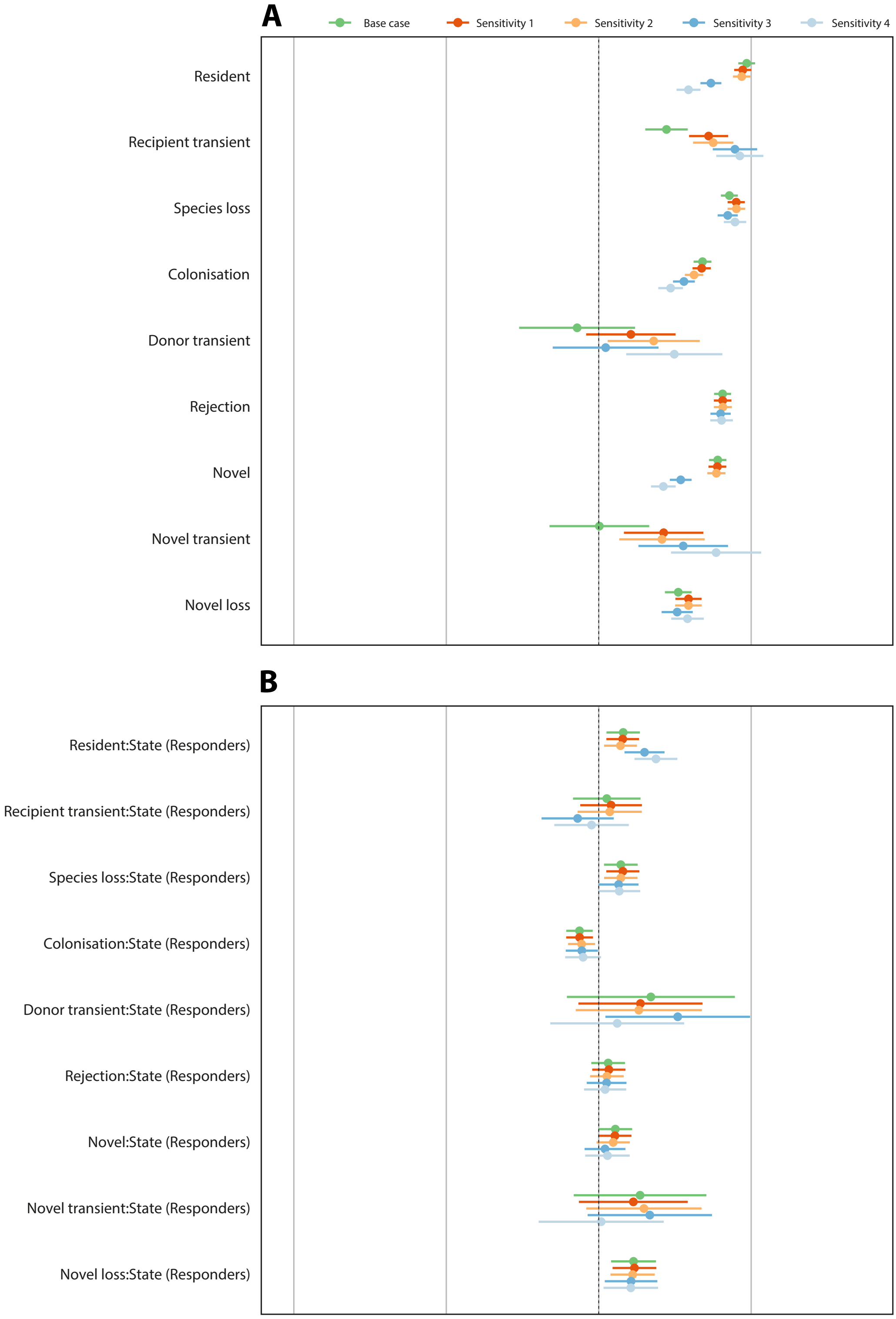

Continues on next page

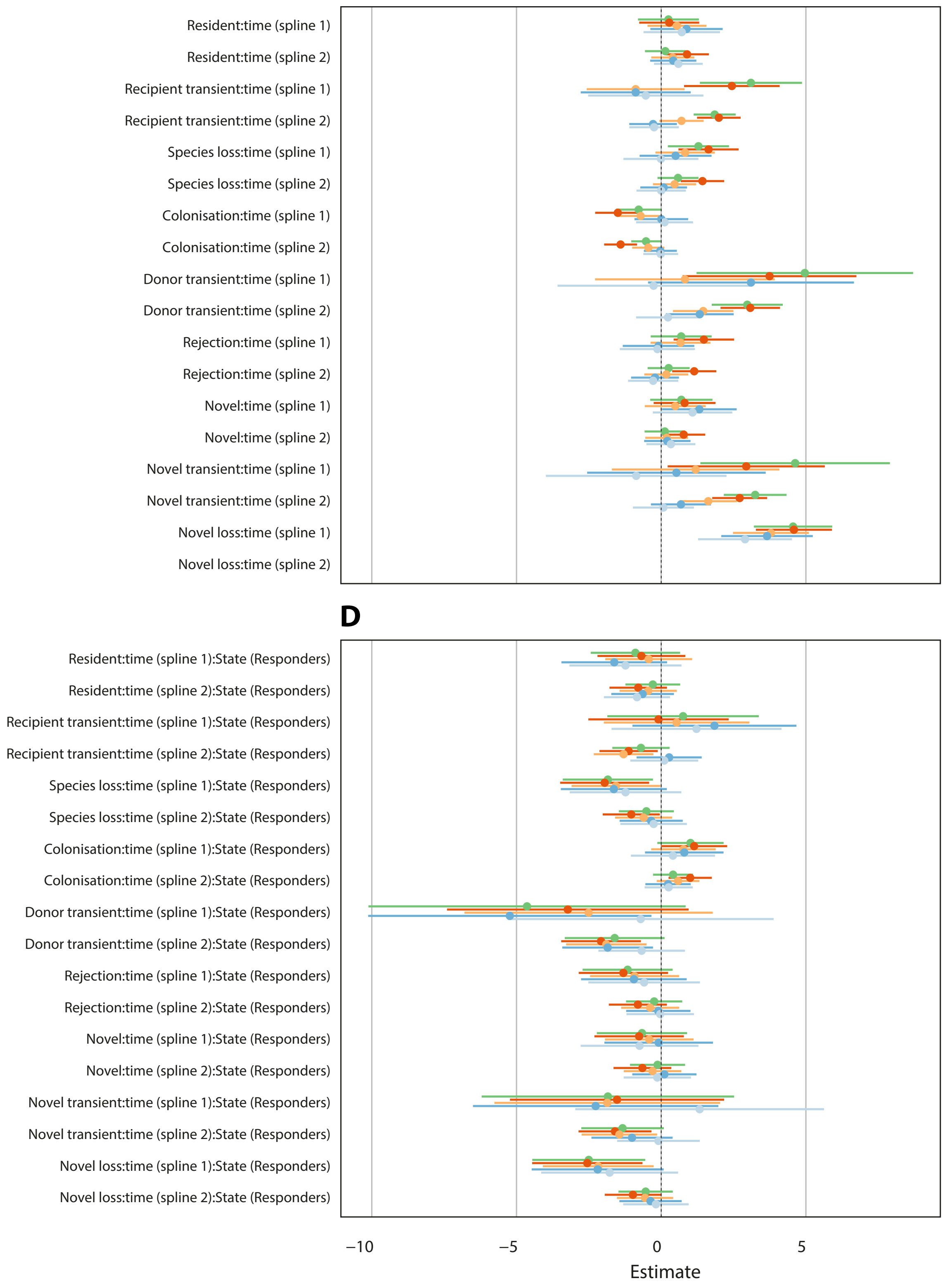

Supplementary Figure S14. Distribution of the number of species per ecological category for the base case and sensitivity (Sensitivity 1 to 4) analyses, estimated by overdispersed Poisson regression models with random effects and splines. *The models contain random intercepts per patient to account for repeated measurements. The point estimates, 95% confidence intervals and a reference line at 0 are shown. When the horizontal lines do not cross the vertical reference line, the coefficients are significantly different from 0. A - D) Model output is presented for variables grouped into four categories for clarity.*
