# Supplementary material for: Ecological resilience in ulcerative colitis: microbial dynamics of donor and resident species in a longitudinal fecal microbiota transplantation study
Source: ISME Commun. 2025 Jul 16;5(1):ycaf119. doi: 10.1093/ismeco/ycaf119 (PMC12378841; doi:10.1093/ismeco/ycaf119)
Supplement: Supplementary_Figure_S15_ycaf119 [file supplementary_figure_s15_ycaf119.pdf]

A

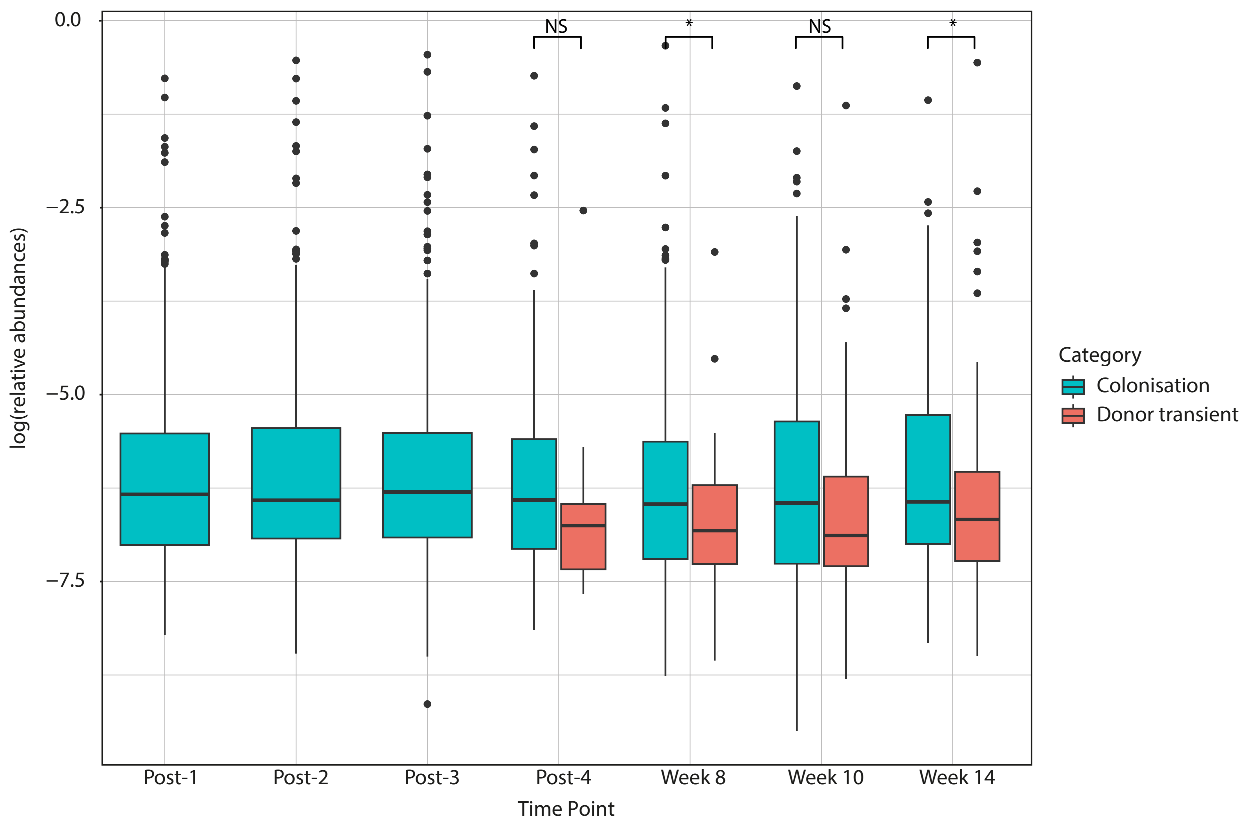

B

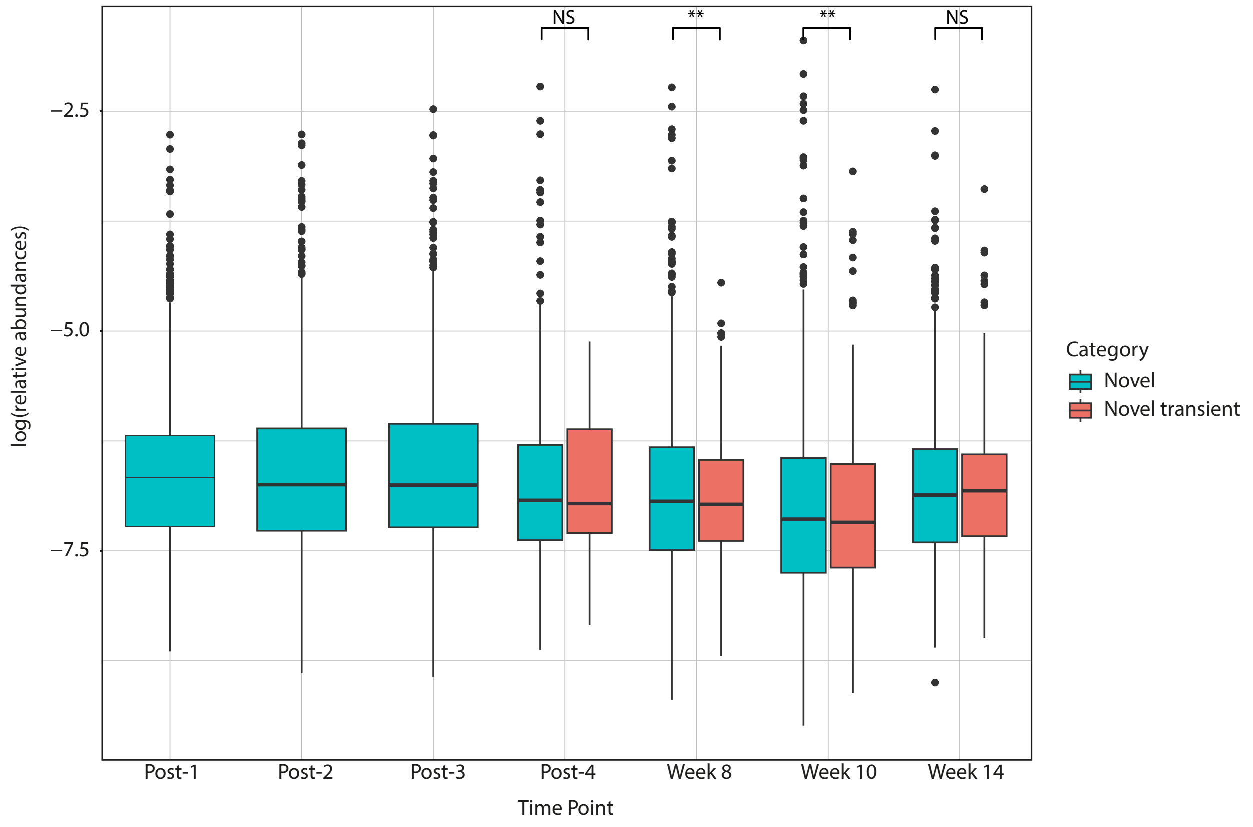

Supplementary Figure S15. Relative abundances of (A) donor and (B) novel species over time. *Results reveal the distribution of abundance differences at particular timepoints across subjects per ecological category for the species that were categorised as donor species and novel species. Significance was tested with linear mixed-models and shown in the plots (*** = p < 0.01; ** = p < 0.01; * = p < 0.05; NS = not significant).*
