# Supplementary material for: Ecological resilience in ulcerative colitis: microbial dynamics of donor and resident species in a longitudinal fecal microbiota transplantation study
Source: ISME Commun. 2025 Jul 16;5(1):ycaf119. doi: 10.1093/ismeco/ycaf119 (PMC12378841; doi:10.1093/ismeco/ycaf119)
Supplement: Supplementary_Figure_S16_ycaf119 [file supplementary_figure_s16_ycaf119.pdf]

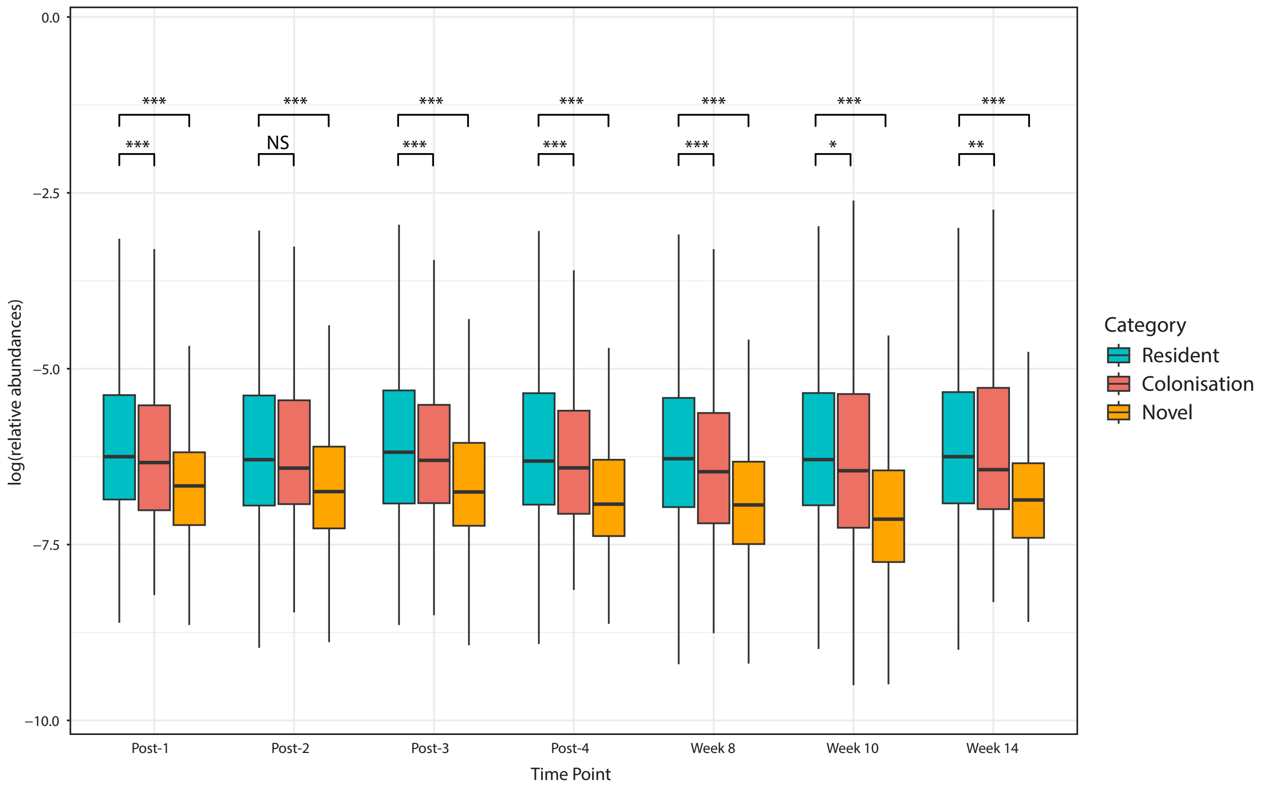

Supplementary Figure S16. Comparison of relative abundance of resident, colonising, and novel species over time. *Significance was tested with linear mixed-models and shown in the plots (*** = p < 0.01; ** = p < 0.01; * = p < 0.05; NS = not significant).*
