# Supplementary material for: Ecological resilience in ulcerative colitis: microbial dynamics of donor and resident species in a longitudinal fecal microbiota transplantation study
Source: ISME Commun. 2025 Jul 16;5(1):ycaf119. doi: 10.1093/ismeco/ycaf119 (PMC12378841; doi:10.1093/ismeco/ycaf119)
Supplement: Supplementary_Figure_S18_ycaf119 [file supplementary_figure_s18_ycaf119.pdf]

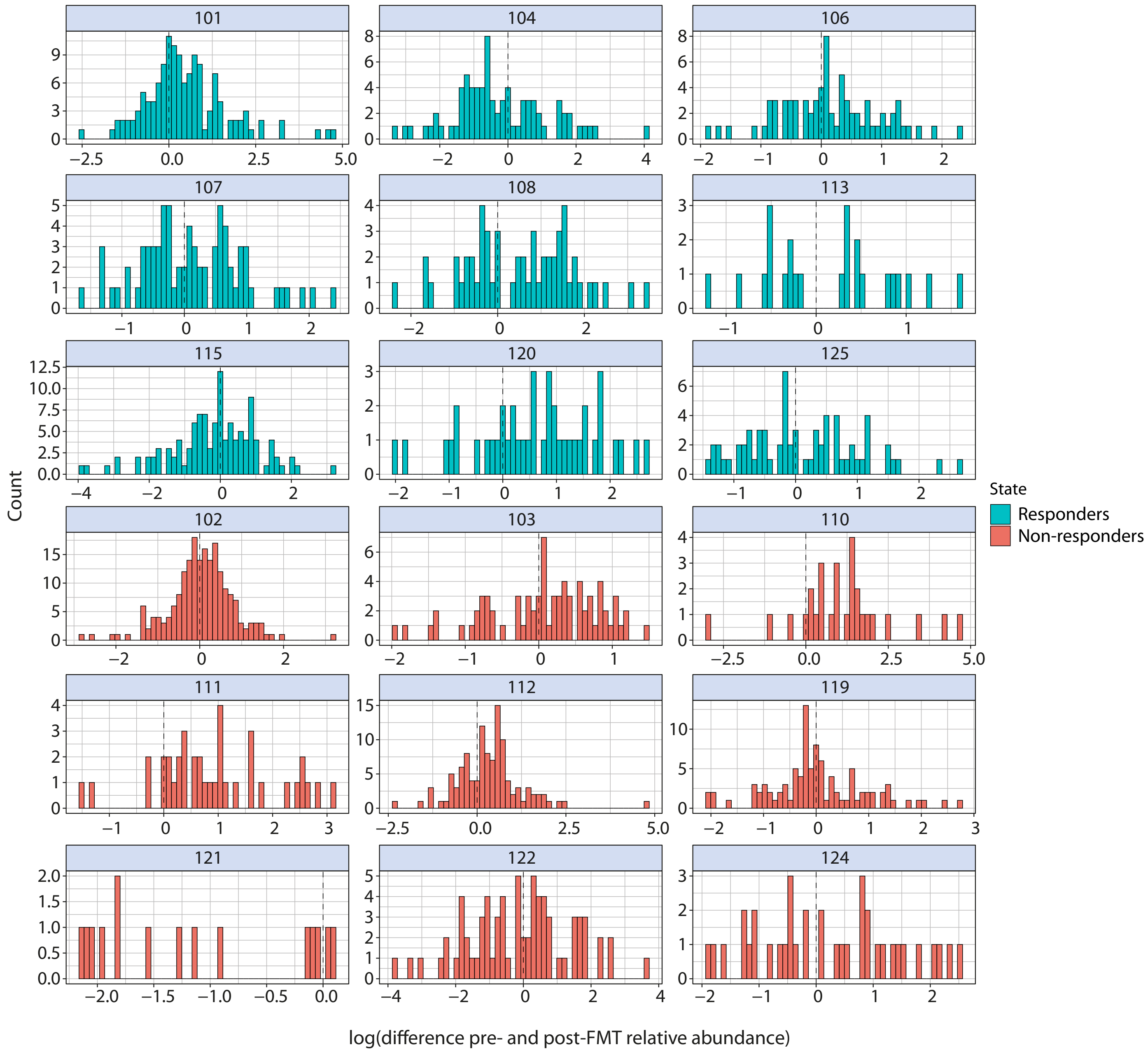

Supplementary Figure S18. Histograms showing the distribution of the differences in relative abundances (between pre- and post-FMT) of resident species. *Only patients that completed the treatment and had at least one post-FMT sample are included in this plot. The striped vertical line indicates no change in abundance between pre- and post-FMT. Because the data had skewed distributions, we used a natural-log transformation of the abundances to normalize the data and homogenize the variance.*
