# Supplementary material for: Ecological resilience in ulcerative colitis: microbial dynamics of donor and resident species in a longitudinal fecal microbiota transplantation study
Source: ISME Commun. 2025 Jul 16;5(1):ycaf119. doi: 10.1093/ismeco/ycaf119 (PMC12378841; doi:10.1093/ismeco/ycaf119)
Supplement: Supplementary_Table_S1_ycaf119 [file supplementary_table_s1_ycaf119.pdf]

Supplementary Table S1. Clinical and demographic information of responders and non-responders.

	**Responders***	**Non-responders****
	Percentage (Number)	*Percentage (Number)*
**Patients**	38% (9)	63% (15)
**Samples**	45% (81)	55% (99)
**Missing**	0	36
**Sex**		
**Female**	67% (6)	40% (6)
**Pretreatment**		
**Budesonide**	56% (5)	53% (8)
**Donor**		
**D07**	22% (2)	67% (10)
	Mean (SD)	*Mean (SD)*
**Age**	48 (16)	45 (17)

* Remission (i.e., response) was defined at week 14 as no symptoms (partial MAYO score of 2 with no individual sub score of >2) and an endoscopic MAYO score 0-1.
** All other patients, including those with a partial response (a decrease of at least 3 points in the partial Mayo score and at least 1 point at the endoscopic Mayo score) at week 14 and patients who left te study early, were classified as non-responders.
