# Supplementary material for: Ecological resilience in ulcerative colitis: microbial dynamics of donor and resident species in a longitudinal fecal microbiota transplantation study
Source: ISME Commun. 2025 Jul 16;5(1):ycaf119. doi: 10.1093/ismeco/ycaf119 (PMC12378841; doi:10.1093/ismeco/ycaf119)
Supplement: Supplementary_Table_S2_ycaf119 [file supplementary_table_s2_ycaf119.pdf]

Supplementary Table S2. Model estimates and p-values for the differences in relative abundances. *Results are also visualised in Figure 3. Multiple linear mixed-effects models were used to test the differences. Significant results are given in red.*

Term	Estimate	Std. error	t-value	p-value
A) Abundance differences per timepoint				
Post-3				
Intercept	-5.72735	0.15259	-37.535	<2e-16
Recipient transient	-0.68310	0.08039	-8.497	<2e-16
Post-4				
Intercept	-5.91299	0.10204	-57.95	<2e-16
Recipient transient	-0.83215	0.06461	-12.88	<2e-16
Week 8				
Intercept	-5.95367	0.10251	-58.08	<2e-16
Recipient transient	-0.83115	0.05676	-14.64	<2e-16
Week 10				
Intercept	-5.75390	0.18769	-30.66	3.73e-10
Recipient transient	-0.84484	0.06146	-13.75	<2e-16
Week 14				
Intercept	-5.90331	0.09321	-63.33	<2e-16
Recipient transient	-0.78173	0.05636	-13.87	<2e-16
B) Relative abundance pre-FMT				
Categories within responders				
Intercept	-5.48590	0.19461	-28.189	2.17e-09
Recipient transient*	-0.63418	0.07692	-8.245	3.91e-16
Species loss*	-0.71556	0.06999	-10.224	< 2e-16
Categories within non-responders				
Intercept	-5.60069	0.18367	-30.49	5.3e-10
Recipient transient*	-0.72155	0.07792	-9.26	< 2e-16
Species loss*	-0.80577	0.06514	-12.37	< 2e-16
Differences in Resident species between responders and non-responders				
Intercept	-5.5100	0.1708	-32.26	1.06e-15
State (non-responders)	-0.1314	0.2432	-0.54	0.597
Differences in Recipient transient species between responders and non-responders				
Intercept	-6.1581	0.1968	-31.299	1.99e-15
State (non-responders)	-0.2003	0.2797	-0.716	0.484
Differences in Species loss species between responders and non-responders				
Intercept	-6.1906	0.2184	-28.348	4.9e-15
State (non-responders)	-0.1883	0.3053	-0.617	0.546
C) Ratio relative recipient species abundances between responders and non-responders				
Intercept	0.191253	0.150087	1.274	0.225
State (non-responders)	-0.006944	0.213633	-0.033	0.9740738

* The difference between the recipient transient and species loss categories for responders and non-responders was tested in a separate model and was not significant (*p*-values were 0.303 and 0.343 for responders and non-responders, respectively).
