# Supplementary material for: Ecological resilience in ulcerative colitis: microbial dynamics of donor and resident species in a longitudinal fecal microbiota transplantation study
Source: ISME Commun. 2025 Jul 16;5(1):ycaf119. doi: 10.1093/ismeco/ycaf119 (PMC12378841; doi:10.1093/ismeco/ycaf119)
Supplement: Supplementary_Information_S1_ycaf119 [file supplementary_information_s1_ycaf119.pdf]

Supplementary Information S1. Clinical and laboratory procedures.

Written informed consent was obtained from all study participants prior to their participation. The patients were included in the study if they had a full MAYO score of 4-9 and a colonoscopy with a MAYO endoscopic sub score of 1-2 within four weeks before study entry. Patients were excluded from this study if they had used antibiotics (<6 weeks), used oral corticosteroids (<8 weeks), surgical treatment (<12 weeks), treatment with any investigational drug in another trial (<12 weeks), significant signs of active infectious gastro-enteritis or enterocolitis, or any other significant medical illnesses. During the study, the medication and diet of the patients was not changed.

Patients randomly received daily treatment for three weeks with either 9 mg budesonide or a placebo drug (Supplementary Table S1). One day before the first FMT a bowel lavage with two liters of Kleanprep (macrogol solution) was performed to cleanse the intestine. Before every fecal transplantation the patients did not eat for at least six hours. The fecal donor suspensions were provided by the Netherlands Donor Feces Bank (NDFB). Collected donor fecal samples were stored and prepared at the LUMC following standard protocols.[1] The NDFB stores donor fecal suspensions for FMT for up to two years.[1] FMTs were infused into the duodenum via a nasoduodenal tube or gastroscope, following standard protocols. Further details on the study population and clinical characteristics are provided by van Lingen et al. (2024).[2]

Each patient was randomly assigned to one of the two donors (D07 and D08), with each donor providing transplants to 12 patients. Patients received all four FMTs from the same donor, though the FMTs were prepared from different donations. Donor samples used for sequencing (n = 13 for donor D07 and n = 14 for donor D08) were part of the actual FMT process. Donor D07 first donated on 14 June 2017 and last donated on 6 March 2019 and donor D08 first donated on 28 August 2017 and lastly on 25 January 2019.

At the end of the study, at week 14, a sigmoidoscopy was performed to assess the endoscopic MAYO score. Remission (i.e., response) was defined at week 14 as no symptoms (partial MAYO score of 2 with no individual sub score of >2) and an endoscopic MAYO score 0-1. Partial remission was defined as a decrease of at least 3 points at the partial Mayo score and at least 1 point at the endoscopic Mayo score. A total of nine patients achieved remission, and one patient achieved partial remission. Of the 14 non–responders, 10 patients left the study early because their symptoms worsened. In total 2 patients did not finish all 4 FMT treatments, these two patients were excluded from further analyses. For this study, we defined a responder as a patient in remission after FMT (n = 9). Non-responders were defined as having activity despite FMT (non-responders and partial responders, n = 15).

DNA was extracted from the donor and recipient stool samples and shotgun sequenced with 100 bp single-end reads to a median depth of 2.9 million reads by Diversigen (New Brighton, Minneapolis, USA) using the Illumina NovaSeq platform. Raw reads mapping to the human genome were removed using bowtie2 (version 2.4.2)[3] and the GRCh37 reference genome and reads were quality-trimmed using fastq (version 0.20.1)[4], both of which are part of an in-house workflow (https://git.lumc.nl/snooij/metagenomics-preprocessing). Unassigned, human-derived, archaeal, and low-quality reads were removed from the data.

References

1. Terveer EM, Vendrik KE, Ooijevaar RE, Lingen EV, Boeije-Koppenol E, Nood EV, et al. Faecal microbiota transplantation for *Clostridioides difficile* infection: Four years' experience of the Netherlands Donor Feces Bank. United European Gastroenterol J. 2020;8(10):1236-1247.
2. van Lingen E, Nooij S, Terveer E, Crossette E, Prince A, Bhattarai S, et al. Fecal Microbiota Transplantation engraftment after budesonide or placebo in patients with active ulcerative colitis using pre-selected donors: a randomized pilot study. J Crohns Colitis. 2024;jjae043:1381-1393.
3. Langmead B, Salzberg SL. Fast gapped-read alignment with Bowtie 2. Nat Methods. 2012;9(4):357-359.
4. Chen S, Zhou Y, Chen Y, Gu J. fastp: an ultra-fast all-in-one FASTQ preprocessor. Bioinformatics. 2018;34(17):i884-i890.
