# Supplementary material for: Ecological resilience in ulcerative colitis: microbial dynamics of donor and resident species in a longitudinal fecal microbiota transplantation study
Source: ISME Commun. 2025 Jul 16;5(1):ycaf119. doi: 10.1093/ismeco/ycaf119 (PMC12378841; doi:10.1093/ismeco/ycaf119)
Supplement: Supplementary_Information_S2_ycaf119 [file supplementary_information_s2_ycaf119.pdf]

Supplementary Information S2. Examples illustrating the categorisation of the species in the base case and in the four sensitivity analyses.

Sensitivity analyses

In Sensitivity 1 we did not allow the occurrence of any single absence when categorising species as lost or as transient upon re-detection (in either the recipient, donor, or novel categories). Secondly, in Sensitivity 2 we only considered the previous timepoint instead of all previous timepoints (Supplementary Figure S1). Therefore, the species can switch more frequently between ecological categories. In Sensitivity 3 and in Sensitivity 4, we considered the full timeseries (also future points) before assigning them to a category with and without considering a single absence respectively (Supplementary Figure S2).

Species present in the recipient pre-FMT

In the base case scenario, a recipient species was present in one of the pre-FMT samples of the recipient. The resident species has been present up to a specific timepoint, however, we have ignored a single absence of the species. If the species was absent for two or more timepoints up to the current one, the species was categorised as a recipient transient species. The third possible category for a recipient species is based on the absence of the species at a specific timepoint and is called 'Species loss'.

Example 1: A species present in the recipient pre-FMT can be categorised as Resident (Res), Recipient transient (RT), or Species loss (SL).

			**Timepoint**						
	Donor	Recipient pre-FMT	1	2	3	4	8	10	14
	Absent	Present	Present	Present	Absent	Present	Present	Present	Present
A – Base case			Res	Res	NA	Res	Res	Res	Res
B – Sensitivity 1			Res	Res	SL	RT	RT	RT	RT
C – Sensitivity 2			Res	Res	SL	RT	Res	Res	Res
D – Sensitivity 3			Res	Res	NA	Res	Res	Res	Res
E – Sensitivity 4			RT	RT	SL	RT	RT	RT	RT

Species identified both in the recipient pre-FMT and the donor are categorized as recipient species into the groups: Resident (Res), Recipient transient (RT), and Species loss (SL) (Example 6.2).

Example 2: A species both present in the recipient pre-FMT and in the donor will be categorised like a recipient species into: Resident (Res), Recipient transient (HT), or Species loss (SL).

			**Timepoint**						
	Donor	Recipient pre-FMT	1	2	3	4	8	10	14
	Present	Present	Absent	Absent	Present	Absent	Absent	Present	Present
A – Base case			NA	SL	RT	SL	SL	RT	RT
B – Sensitivity 1			SL	SL	RT	SL	SL	RT	RT
C – Sensitivity 2			SL	SL	RT	SL	SL	RT	Res
D – Sensitivity 3			NA	SL	RT	SL	SL	RT	RT
E – Sensitivity 4			SL	SL	RT	SL	SL	RT	RT

Donor derived species

A donor species is a species that was not detected in the recipient pre-FMT, and that was present in the core donor microbiota. Again, there are three possible categories: Colonisation, Donor transient, and Rejection. Species are categorised to rules similar to how the recipient species are categorised (Colonisation similar to Resident, Donor transient similar to Recipient transient, and Rejection similar to Species loss). However, a species can still be placed in the Colonisation category after being absent (i.e., Rejection) for some timepoints, as it is possible that a species does not colonise directly after the first FMT, but that it needs time to establish in the gut. Species can also be categorized in the Rejection category even after being absent for some timepoints.

Similar to other categories, this occurs when a species initially colonises but later disappears. Over time, if multiple species follow this pattern, the number of rejected species increases. Note, that also in this category a species is allowed and ignored if it is absent once, but only after being present.

Example 3: A species not present in the recipient pre-FMT, but present in the donor can be categorised as Colonisation (C), Donor transient (DT), or Rejection (Rej).

			**Timepoint**						
	Donor	Recipient pre-FMT	1	2	3	4	8	10	14
	Present	Absent	Present	Present	Absent	Present	Present	Absent	Present
A – Base case			C	C	NA	C	C	Rej	DT
B – Sensitivity 1			C	C	Rej	DT	DT	Rej	DT
C – Sensitivity 2			C	C	Rej	DT	C	Rej	DT
D – Sensitivity 3			DT	DT	NA	DT	DT	Rej	DT
E – Sensitivity 4			DT	DT	Rej	DT	DT	Rej	DT

Novel species

A novel species has not been present or was under the detection limit in the pre-FMT recipient samples, as well as in the core donor microbiota. Like colonising species, also novel species can enter the microbiota of the recipient later. However, where a donor species is in that case categorised as 'Rejected', the novel species is not categorised as 'Novel loss', but as 'Absent' and not taken into account in the analyses, until the species has been present once.

Example 4: A species not present in either the recipient pre-FMT or the donor can be categorised as Novel (N), Novel transient (NT), or Novel lost (NL), from the moment the species appeared in the patient samples.

			**Timepoint**						
	Donor	Recipient pre-FMT	1	2	3	4	8	10	14
	Absent	Absent	Absent	Present	Present	Present	Absent	Present	Absent
A – Base case			-	N	N	N	NA	N	NL
B – Sensitivity 1			-	N	N	N	NL	NT	NL
C – Sensitivity 2			-	N	N	N	NL	NT	NL
D – Sensitivity 3			-	NT	NT	NT	NA	NT	NL
E – Sensitivity 4			-	NT	NT	NT	NL	NT	NL
